# Detoxification of Indole by an Indole-Induced Flavoprotein Oxygenase from *Acinetobacter baumannii*

**DOI:** 10.1371/journal.pone.0138798

**Published:** 2015-09-21

**Authors:** Guang-Huey Lin, Hao-Ping Chen, Hung-Yu Shu

**Affiliations:** 1 Microbial Genetics Laboratory, Department of Microbiology, Tzu-Chi University, Hualien, Taiwan; 2 Department of Biochemistry, School of Medicine, Tzu-Chi University, Hualien, Taiwan; 3 Department of Bioscience Technology, Chang Jung Christian University, Tainan, Taiwan; Dong-A University, REPUBLIC OF KOREA

## Abstract

Indole, a derivative of the amino acid tryptophan, is a toxic signaling molecule, which can inhibit bacterial growth. To overcome indole-induced toxicity, many bacteria have developed enzymatic defense systems to convert indole to non-toxic, water-insoluble indigo. We previously demonstrated that, like other aromatic compound-degrading bacteria, *Acinetobacter baumannii* can also convert indole to indigo. However, no work has been published investigating this mechanism. Here, we have shown that the growth of wild-type *A*. *baumannii* is severely inhibited in the presence of 3.5 mM indole. However, at lower concentrations, growth is stable, implying that the bacteria may be utilizing a survival mechanism to oxidize indole. To this end, we have identified a flavoprotein oxygenase encoded by the *iifC* gene of *A*. *baumannii*. Further, our results suggest that expressing this recombinant oxygenase protein in *Escherichia coli* can drive indole oxidation to indigo *in vitro*. Genome analysis shows that the *iif* operon is exclusively present in the genomes of *A*. *baumannii* and *Pseudomonas syringae* pv. *actinidiae*. Quantitative PCR and Western blot analysis also indicate that the *iif* operon is activated by indole through the AraC-like transcriptional regulator IifR. Taken together, these data suggest that this species of bacteria utilizes a novel indole-detoxification mechanism that is modulated by IifC, a protein that appears to be, at least to some extent, regulated by IifR.

## Introduction

More than 85 species of bacteria produce indole, an aromatic organic compound that is known to act as an extracellular signaling and regulatory molecule in a variety of physiological processes [1, 2]. Tryptophanase, encoded by the *tnaA* gene, is a tryptophan indole-lyase that produces indole, ammonium, and pyruvate from the amino acid tryptophan in *Escherichia coli* [3, 4]. Notably, the tryptophanase operon, encoding both tryptophanase and tryptophan permease, is regulated by glucose and tryptophan [5, 6]. For example, *E*. *coli* cultured in lysogeny broth (LB) media can secrete up to 0.5 mM of indole in the stationary phase, but when cultured in medium containing excess tryptophan, the indole concentration can reach up to 5 mM in the media [7]. Further, this increased concentration of indole is even higher inside the cell compared to the extracellular media as indole has a high affinity for lipids and will transverse the hydrophobic membrane of the cell [8] allowing this diffusible signaling molecule to regulate gene expression and numerous downstream processes, including fungal and bacterial growth.

During cellular growth, indole appears to exhibit both oxidant toxicity [9] and proton ionophoric activity [8, 10], which modulate the inhibition of cell division. Further, this inhibition can be induced by very low concentrations of indole. For example, in *Aspergillus niger*, just 0.43 mM of indole is sufficient to inhibit growth [11]. Similarly, a low level of indole (20 μg/ml; 0.17 mM) also inhibits the growth of four lactic acid bacterial strains known to function in the human intestine [12]. In fact, some bacteria in the intestinal tract are known to secrete indole or indole derivatives in order to inhibit the growth of other bacteria [2], thereby eliminating competition in their environment. Thus, it is not surprising that these compounds can greatly affect the host’s immune system as small changes in concentration can result in major fluctuations in the composition of gut microbiota [2, 13].

Notably, several types of bacterial, plant, and animal species have developed enzymatic defense systems to interfere with indole signaling and overcome indole-induced oxidant toxicity [2]. For instance, *E*. *coli* expressing wild-type and mutant cytochrome P450 enzymes from mammalian, human, and bacterial sources can convert indole into non-toxic indigo and indigoids [14–20]. In a similar manner, many bacterial species, including *Acinetobacter* spp., have been shown to transform indole to indigo in the presence of an aromatic inducer, such as phenol and aromatic hydrocarbons [21, 22]. Furthermore, recombinant *E*. *coli* expressing exogenous genes from this species also convert indole to various indigoids [21, 23]. While research to identify the species specific genes responsible for this enzymatic defense against indole toxicity is ongoing, the full function and regulation of these genes is largely unknown.

*A*. *baumannii* is a gram-negative bacterial pathogen found in human feces [24] and the rhizosphere [25]. In these environments, *A*. *baumannii* may encounter indole produced by other living organisms as well as industrial sources [26]. However, it is unclear whether the growth of *A*. *baumannii* is affected by indole. In the present study, we have shown that high concentrations of indole disrupt *A*. *baumannii* growth. However, at low indole concentrations, this bacterial species appears to utilize an enzymatic defense against this toxic compound. Furthermore, we have also characterized the function and regulation of an oxygenase encoded by the *iif* (*iif* stands for indole induced flavoprotein) operon, which appears to be involved in the oxidation and detoxification of indole in this species of bacteria.

## Materials and Methods

### Bacterial growth conditions and primers

*A*. *baumannii* and *E*. *coli* strains were routinely grown in LB medium or LB agar [27] at 37°C with vigorous shaking. M9 minimal medium (Amresco, Solon, OH) was used for the indole toxicity test. Ampicillin, chloramphenicol, and kanamycin were added to the medium when needed at 100 μg/ml, 12.5 μg/ml, and 50 μg/ml, respectively. Primers used in this study are listed in Table 1.

**Table 1:** Primers used in this study.

Primer	Sequence^a^	Gene/direction
**Cloning primers**		
OxyF	5′-GCAGCCGATTATCACTTACTAGGCCG-3′	
OxyR	5′-CTCTTGGGTTTTCGGCATTAATCGC-3′	
IifCF	5′-GCATGGATCCATGCGCCGTATTGCAATTGTTG-3′	
IifCR	5′-GCATCTGCAGGACCTTAGGCCACTTTTGCTGTG-3′	
BslF	5′-GCCTCACTGATTAAGCATTGG-3′	
BslR	5′-CATCAGAGCAGCCGATTGTCTG-3′	
IifRF	5′-CACCGCCAATGATTGAAGCCG-3′	
IifRR	5′-CCTACTGGCGAATACGACCAG-3′	
cIifRF	5′-GGATCCAGAGCAATACCTCACGCTAAAAG-3′	
cIifRR	5′-GGATCCTGCAAGTTCGCTATGTTCACC-3′	
cIifPF	5′-GGATCCTTGGTAATTCCCCAATCATCC-3′	
cIifPR	5′-CCTAGTAAGTGATAATCGGCTGCCCTGACATTGTTATGGCTTTATCTTCTATC-3′	
cIifCF	5′- GATAGAAGATAAAGCCATAACAATGTCAGGGCAGCCGATTATCACTTACTAGG -3′	
cIifCR	5′-GGATCCGACCTTAGGCCACTTTTGCTGTG-3′	
**RT-PCR primers**		
IifF1	5′-TTAGACCCAACAGGCTTACCAGTTG-3′	*iifA*/forward
IifR1	5′-CAATCGCCTTACCTTTGGCATC-3′	*iifB*/reverse
IifF2	5′-TCATCCCGAAGAAGTCGCCAATG-3′	*iifB*/forward
IifR2	5′-GATTTCATCGGCAGTACGGTTG-3′	*iifC*/reverse
IifF3	5′-GCCAATAACTTTGATGACCCGC-3′	*iifC*/forward
IifR3	5′-AATGGCGGATCTAAAGACAGAGATG-3′	*iifD*/reverse
IifF4	5′-AGTATGAAGGCGGAGATCACCTG-3′	*iifD*/forward
IifR4	5′-CGGCAAACCTAATAATGTCCCTGC-3′	*iifE*/reverse
**RT-qPCR primers**		
qF911_02007F	5′-TAGGTTTAGGCGCAGCTATGCC-3′	F911-02007^b^/forward
qF911_02007R	5′-ATCACTTTGGCCCAGCCATAGC-3′	F911-02007^b^/reverse
qIifAF	5′-GCACGACCAAAAAGCAACCATTAC-3′	*iifA*/forward
qIifAR	5′-CATGACGGATATGCTCTTCCCAG-3′	*iifA*/reverse
qIifBF	5′-CTGAGCCGTGCTTTATACAATGACC-3′	*iifB*/forward
qIifBR	5′-GAAACCGAATTGACGCGAATACC-3′	*iifB*/reverse
qIifCF	5′-CAACCGTACTGCCGATGAAATC-3′	*iifC*/forward
qIifCR	5′-AGCCCAATGCCTTCTACGGC-3′	*iifC*/reverse
qIifDF	5′-GGCCGGAAAATAGGCATGAC-3′	*iifD*/forward
qIifDR	5′-AAAATGCCCCGAAAGCTGATG-3′	*iifD*/reverse
qIifEF	5′-GTATTGGGCTGCCACATATTGG-3′	*iifE*/forward
qIifER	5′-GCCCAGCTTGCATATCATTTGC-3′	*iifE*/reverse
qIifRF	5′-TGAACCAAGCCAACTCCCAC-3′	*iifR*/forward
qIifRR	5′-GCCTACATTTCGGCAGGTTTC-3′	*iifR*/reverse
qGyrBF	5′-GGCGGCTTATCTGAGTTTGT-3′	*gyrB*/forward
qGyrBR	5′-TTTGTGGAATGTTATTTGTG-3′	*gyrB*/reverse

^a^Restriction recognition sites are underlined.

^b^F911-02007 is the locus_tag for the gene located immediately upstream *iifA*.

### Indole and indigo toxicity test

To test the effects of different concentrations of indole on the growth of wild-type *A*. *baumannii*, 1 ml of overnight LB culture media was collected and centrifuged. The supernatant was discarded and the pellet was resuspended in 500 μl of M9. This aliquot was then transferred to a flask containing 50 ml of M9 and incubated. Thus, after adding varying amounts of indole (dissolved in absolute ethanol) (0.5–5 mM), the culture was incubated at 37°C, 200 rpm for 16 h. The final concentration of ethanol was 0.1% and ethanol acted as the sole carbon source in this environment. The optical density at 600 nm (OD_600_) was used to determine the growth of the bacteria under the various conditions. Experiments were performed in triplicate.

To test the toxicity of indigo, the experimental culture condition was conducted as described above. After adding indole or indigo (5 mM), the culture was incubated at 37°C, 200 rpm for 16 h. The drop plate counting method was used to count the total number of bacteria [28]. The experiment was carried out three times.

Next, we compared the effects of 3 mM indole on the growth of the *iifC* and *iifR* mutants, described in the following sections, to the wild-type *A*. *baumannii* using the methods described above. Notably, the growth rate was measured every 2 h using the OD_600_. Experiments were performed three times.

### Screening of indigo-forming *E*. *coli* colonies and identification of functional genes

We previously constructed a fosmid library representing 10-fold coverage of the *A*. *baumannii* ATCC19606 genome [23]. To isolate blue indigo-forming fosmid colonies, individual clones from the fosmid library were cultured on LB plates containing chloramphenicol overnight at 37°C, followed by incubation at 4°C for 7 days. In doing so, the indigo-producing fosmid, pOXY, was identified. pOXY was then purified and the insert end sequences were determined by sequencing as previously described [23].

### Sequence analysis

BLAST algorithm (http://blast.ncbi.nlm.nih.gov/Blast.cgi), MacVector software [29], and Pfam [30] were used to search the GenBank database, align sequences, and search the protein domains, respectively. The helical regions of proteins were predicated by YASPIN [31].

### Quantitative reverse transcription PCR (RT-qPCR) and reverse transcription PCR (RT-PCR)

Gene expression was investigated using RT-qPCR. For RNA purification, we collected 1 ml of *A*. *baumannii* overnight LB culture media for centrifugation. The supernatant was then discarded and cells were resuspended in 500 μl M9. The resuspended cells were transferred into 50 ml of M9 containing 1.5 mM indole and 0.1% ethanol and incubated at 37°C, 200 rpm for 6 h. RNA was extracted during the exponential-phase of the cultures using TRIzol reagent (Invitrogen). After resuspension in TRIzol, chloroform was added, and the mixture was centrifuged at 4°C, 12,000 × *g* for 15 min. An equal volume of 70% ethanol was then added to the supernatant and RNA was extracted using the RNeasy Mini Kit (Qiagen, Hilden, Germany) according to the manufacturer’s protocol. The concentration of extracted RNA was determined using a Nanodrop ND-1000 spectrophotometer (Thermo Fisher Scientific Inc.; Wilmington, DE). Aliquots of the samples were analyzed on an RNA 6000 Nano chip in order to measure the RNA integrity using an Agilent 2100 bioanalyzer (Agilent Technologies; Santa Clara, CA). RNA was reverse transcribed using M-MLV reverse transcriptase (Promega) and qPCR was performed in triplicate with a LightCycler 1.5 instrument (Roche Diagnostics; Mannheim, Germany) using the LightCycler FastStart DNA MasterSYBRE Green I kit (Roche). Relative gene expression was calculated as previously described [32], using gyrase subunit B gene (*gyrB*) expression as an internal control. Fold change was determined by the 2(^-ΔΔ^ *C*_T_) method and the graphs represent means ± SD. Primer pairs used for RT-qPCR are listed in Table 1. The following PCR cycling conditions were used: 1 cycle at 95°C for 10 min, 40 cycles at 95°C for 10 s, followed by 60°C for 15 s.

For RT-PCR, Total RNA was purified from *A*. *baumannii* cultured in M9 medium containing 1.5 mM indole using TRIzol and reverse transcribed as described above. The cDNA was amplified by PCR with Herculase II Fusion DNA polymerase (Agilent Technologies) and specific primer pairs for *iifA-iifB*, *iifB-iifC*, *iifC-iifD*, and *iifD-iifE* intergenic regions (Table 1).

### Transposon insertion

To identify the indigo-producing gene in the pOXY clone, the fosmid was purified and pOXY insertion mutants were generated by transposition *in vitro* with the EZ-Tn*5* <KAN-2> transposon (Epicentre) following the manufacturer’s instructions. Transposon-tagged pOXY (pOXY::KAN-2) fosmids were then transformed into *E*. *coli* EPI300 (Epicentre) and selected on LB agar containing chloramphenicol and kanamycin. The transposon-tagged fosmids, which did not produce blue pigment when expressed in *E*. *coli*, were isolated, and the transposon insertion sites were determined with sequencing as previously described [23]. Of these fosmids, we selected nine, all of which appear to insert the KAN-2 transposon into the *iifC* gene (designated pOXY-*iifC*::KAN-2 plasmids).

### *iifC* gene amplification and protein expression vector construction

A DNA fragment containing the indigo-producing gene *iifC* and the 223-bp region upstream of the putative translation initiation site was amplified by PCR using primers (OxyF and OxyR). The amplified DNA fragment was cloned using StrataClone Blunt PCR Cloning Kit (Agilent Technologies, Santa Clara, CA). The PCR products were ligated with Topoisomerase I-charged pSC-B vector arms and transformed into the provided StrataClone SoloPack competent cells according to the manufacturer’s protocol. Transformants were selected on LB agar containing ampicillin. The resulting plasmid was named pSCB-OXY.

To construct the IifC protein expression plasmid, the functional *iifC* gene was first amplified via PCR using the IifCF and IifCR primers. The PCR product was then digested with *Bam*HI and *Pst*I restriction enzymes and ligated into the *Bam*HI/*Pst*I digested pQE-80L expression plasmid (Qiagen) to create the pQE80L-IifC plasmid. This expression plasmid was then transformed into two *E*. *coli* strains: CY15000, a bacterial strain that harbors a mutation in the *tnaA* gene [33] obtained from the *E*. *coli* Genetic Stock Center (CGSC7682); and DH5α competent cells.

### Isolation and characterization of pigments

Thin-layer chromatography (TLC) analysis was used to examine the pigments produced in the *E*. *coli* DH5α(pQE80L-IifC) and *E*. *coli* CY15000(pQE80L-IifC). *E*. *coli* DH5α(pQE80L-IifC) and *E*. *coli* CY15000(pQE80L-IifC) were cultured at 37°C overnight in LB broth. For pigments purification, 500 μl overnight culture was extracted using an equal volume of ethyl acetate. The ethyl acetate layer was separated by centrifugation and then evaporated. The blue pigment was resuspended in ethyl acetate, spotted on a silica gel plate (Silica gel 60 F_254_; catalogue number 05554; Merck), and developed with chloroform-methanol/50-1 (v/v).

### Mutation of the *iifC* and *iifR* genes and complementation testing

Mutation of the *iifC* and *iifR* genes (designed Δ*iifC* and Δ*iifR* strains) was conducted using conjugation-mediated allelic exchange and confirmed with PCR as described in the S1 Methods section. Further, plasmids were also constructed for each mutant to perform complementation testing to determine their functional role in indigo production.

### Indoline oxidization test

Further, in order to determine if indoline can be oxidized by IifC, *E*. *coli* CY15000(pQE80L-IifC) cultures were grown at 37°C overnight in LB broth supplemented with ampicillin and indoline. Following overnight cultivation, changes in the color of the media were monitored.

### Recombinant protein purification and enzymatic activity assay

To produce recombinant IifC protein, *E*. *coli* CY15000(pQE80L-IifC) was cultured in LB at 37°C until an OD_600_ of 0.4 was reached. The recombinant protein was expressed by adding 1 mM isopropyl-β-D-thiogalactopyranoside (IPTG) to the culture. The culture was then incubated with agitation at 20°C for 4 h, after which the cells were pelleted by centrifugation and stored at -20°C until use. Protein expression was examined using 10% sodium dodecyl sulfate-polyacrylamide gel electrophoresis (SDS-PAGE) [34], followed by Coomassie blue G250 staining. The recombinant IifC protein was then purified and enzyme activity was analyzed using previously optimized assay procedures [23]. The flavin adenine dinucleotide (FAD) concentration in purified IifC was determined following the procedure described elsewhere [35].

### Preparation of IifC and IifR polyclonal antibodies and Western blot analysis

Rabbit polyclonal antibodies against the purified recombinant IifC and IifR proteins were generated using a previously documented antiserum purification procedure [23]. Western blot analysis was then performed as previously described [23] with some modifications specific to these novel antibodies. Briefly, bacterial proteins were separated by 8% SDS-PAGE, blotted onto a polyvinylidene fluoride (PVDF) membrane, and blocked with Tris buffered saline (TBS; 50 mM Tris-HCl [pH 7.6], 0.9% NaCl) containing 5% milk for 1 h. The PVDF membrane was then incubated with either anti-IifC (dilution, 1:20,000) or anti-IifR (dilution, 1:25,000), along with a mouse monoclonal antibody against the alpha-subunit of RNA polymerase (dilution, 1:1,000; Neoclone, Madison, WI) for 1 h. After washing the membrane 6 times with TTBS buffer (TBS containing 0.25% Tween 20) for 10 min each, it was incubated with horseradish peroxidase-conjugated goat anti-rabbit IgG (dilution, 1:2,000; Santa Cruz Biotechnology, Dallas, TX) or horseradish peroxidase-conjugated goat anti-mouse IgG (dilution, 1:2,000; Santa Cruz Biotechnology) secondary antibody for 1 h and washed with TTBS buffer 5 times, 10 min each. Protein expression was visualized with enhanced chemiluminescence reagent (Pierce ECL Western Blotting Substrate; Thermo Scientific).

## Results

### Indole toxicity assay

**Fig 1:**
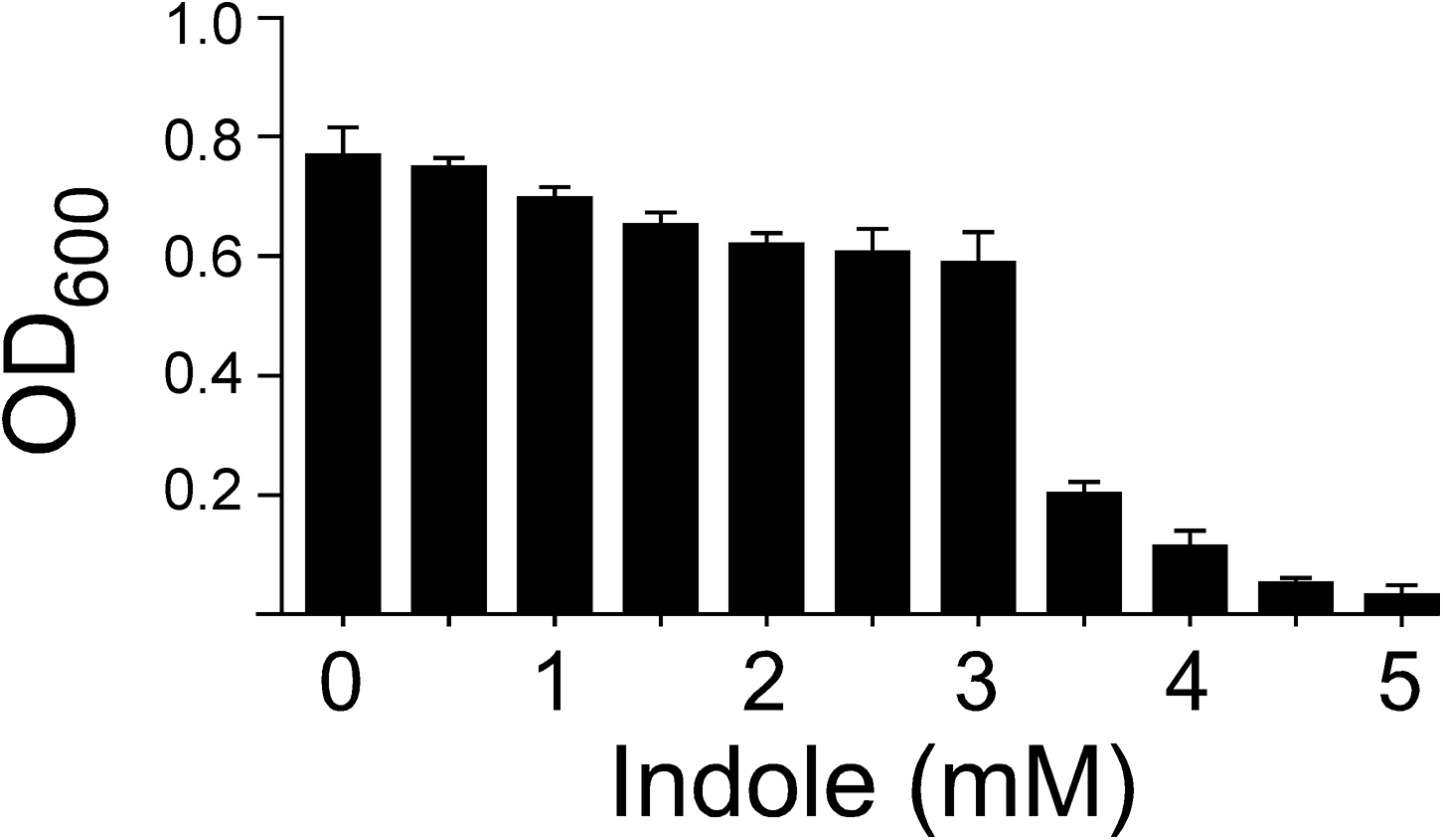
Effect of indole on the growth of wild-type *A*. *baumannii* ATCC 19606. Wild type *A*. *baumannii* ATCC 19606 was grown in M9 medium supplemented with 0–6 mM indole for 16 h.

To determine whether indole inhibits the growth of *A*. *baumannii*, *A*. *baumannii* were cultured in M9 media containing varying concentration of indole (0.5–5 mM). The OD_600_ readings gradually decreased with the increasing indole concentration, with a severe decline in cell density at 3.5 mM (Fig 1). The toxicity assay was used to compare the effects of indole and indigo on bacteria growth. Unlike indole, 5 mM indigo does not inhibit the growth of *A*. *baumannii* (S1 Fig). These data indicate a possible defense against indole toxicity in *A*. *baumannii* when the indole concentration is low. To this end, *A*. *baumannii* ATCC19606 cultured in M9 medium with indole were observed to produce indigo (S2 Fig), suggesting the involvement of an enzymatic indole-conversion pathway.

### Identification of indigo producing genes in *A*. *baumannii*

**Fig 2:**
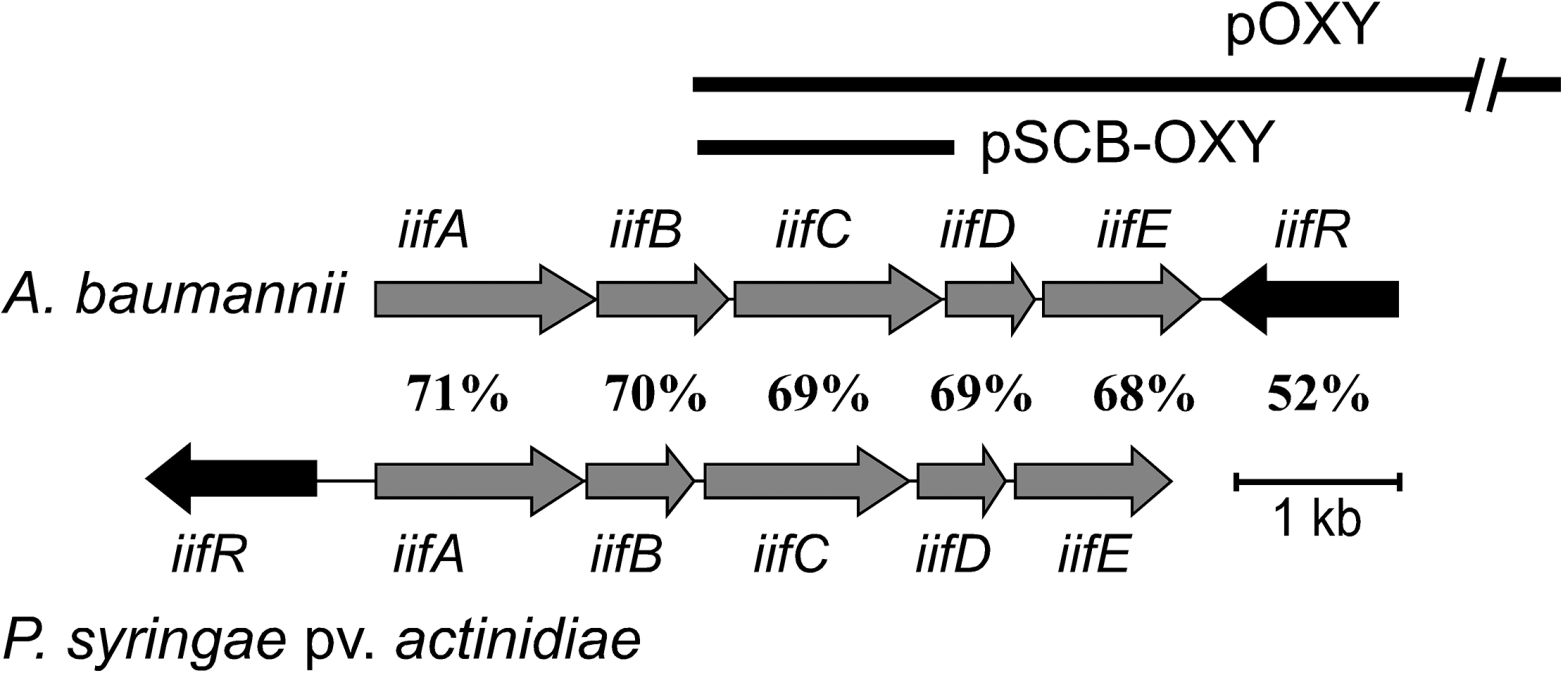
The gene organization of the *iif* operon in *A*. *baumannii* ATCC 19606 and *P*. *syringae* pv. *actinidiae* ICMP 19070. Gray arrows represent the five-gene operon in *A*. *baumannii* ATCC 19606 and the dark-gray arrows represent the AraC-like regulator *IifR*. Thick lines represent the inserts of pOXY and pSCB-OXY. In *A*. *baumannii* ATCC 19606, the locus_tag for *iifA*, *iifB*, *iifC*, *iifD*, *iifE*, and *iifR* is F911_02006, F911_02005, F911_02004, F911_02003, F911_02002, and F911_02001, respectively. In *P*. *syringae* pv. *actinidiae* ICMP 19070, the locus_tag for *iifA*, *iifB*, *iifC*, *iifD*, *iifE*, and *iifR* is A259_30100, A259_30105, A259_30110, A259_30115, A259_30120, and A259_30095, respectively.

A fosmid library of *A*. *baumannii* ATCC19606 was previously constructed [23] and 960 fosmid clones were screened on LB plates in this study. One fosmid clone, pOXY, was consistently observed to produce blue pigments, leading us to isolate this colony. Sequence analysis and paired-end sequence mapping of the clone revealed that pOXY contains a 35,647 bp region of *A*. *baumannii* ATCC19606 genomic DNA, starting at the 130,733th bp and ending at the 166,379th bp of the 13^th^ contig (Accession number APRG01000013) (Fig 2).

### Sequence analysis of the *iif* operon of *A*. *baumannii*

Using RT-PCR to amplify the four putative intergenic regions of the *iif* operon found in *A*. *baumannii*, it appears that these four regions are amplified as a single *iifABCDE* operon in the presence of indole (S3 Fig). Further, this *iif* organization is prevalent in multiple *A*. *baumannii* strains, including ATCC19606, AB0057, AB307-0294, AB900, ACICU, AYCC 17978, and AYE. Aside from these *A*. *baumannii* strains, this *iif* operon is also found in *Pseudomonas syringae* pv. *actinidiae* and the arrangement of the operon in these two species is identical (Fig 2). Further, sequence analysis of the regulatory *iifR* gene indicates that the transcription direction of the gene is opposite to that of those in the main operon in both species; however, the *iifR* gene in *A*. *baumannii* is located downstream of the operon, while it is located upstream of the operon in *P*. *syringae* pv. *actinidiae*. The protein identity between each *iif* gene in *A*. *baumannii* ATCC19606 and *P*. *syringae* pv. *actinidiae* is also shown in Fig 2.

The G+C content of each *iif* gene present in *A*. *baumannii* ATCC19606 and the domains of the protein products encoded by *iifABCDER* are listed in Table 2. Using a Pfam database search, we found that IifA belongs to the dienelactone hydrolase family and shows strong homology (72% identity) to the putative dienelactone hydrolase of *Pseudomonas* sp. GM102 (accession no. EJL95871). Further, IifB appears to be a putative short-chain dehydrogenase. Sequence analysis of the *iifC* gene indicates that it is 1,239 bp long and the ATG start codon is preceded by a TGGAG ribosome-binding site. Although our sequence analysis did not reveal any significant Pfam domains, IifC showed 64.8% (268/413), 59.8% (241/403) and 47.4% (196/413) identity to MoxY (uncultured bacterium; GenBanK accession No. ABQ12175), StyA1 of *Rhodococcus opacus* 1CP (GenBanK accession Nos. ACR43973 and AFO70154), and StyA2B of *R*. *opacus* 1CP (GenBanK accession Nos. ACR43974 and AFO70155), respectively (S4 Fig). IifD showed 67.9% (114/168) identity to the flavin reductase of *Pseudomonas fuscovaginae* (accession No. WP_029379307), while IifE showed 73.6% (217/295) identity to the protein involved in the MetA-pathway of phenol degradation-like protein of *Pseudomonas fuscovaginae* (accession No. WP_019363187). Moreover, sequence analysis of IifR revealed that this protein contains two helix-turn-helix motifs (residues 255 to 332; C-terminal DNA binding domain) and belongs to the AraC/XylS family (S5 Fig). Multi sequence alignment of the DNA binding domain of this family revealed that, like AraC and XylS, IifR of *A*. *baumannii* ATCC19606 and *P*. *syringae* pv. *actinidiae* also contains the consensus binding sequence utilized by the AraC/XylS family [36].

**Table 2:** The *iif* genes of *A*. *baumannii* ATCC19606.

Genes	Locus_tag	G+C content (%)	Product size (aa)	Protein Accession No.	Pfam family (Description)^a^	Pfam number (E-Value)^a^
*iifA*	F911-02006	44.04	416	ENW75089	DLH/Dienelactone hydrolase family	PF01738 (1.1e-49)
*iifB*	F911-02005	45.67	261	ENW75088	Adh_short_C2 (Enoyl-Acyl carrier protein reductase)	PF13561 (3.3e-31)
*iifC*	F911-02004	45.50	412	ENW75087	No significant matches	
*iifD*	F911-02003	42.94	176	ENW75086	Flavin_Reduct (Flavin reductase like domain)	PF01613 (2.2e-33)
*iifE*	F911-02002	40.72	315	ENW75085	Phenol_MetA_deg (Putative) MetA-pathway of phenol degradation	PF13557 (2.2e-63)
*iifR*	F911-02001	37.77	352	ENW75084	AraC_binding_2 (AraC-binding-like domain), HTH_18 (Helix-turn-helix domain)	PF14525 (6.1e-39), PF12833 (2.6e-28)

^a^Determined by searching the Pfam database.

### Identification of *iifC* as an important player in indigo production

**Fig 3:**
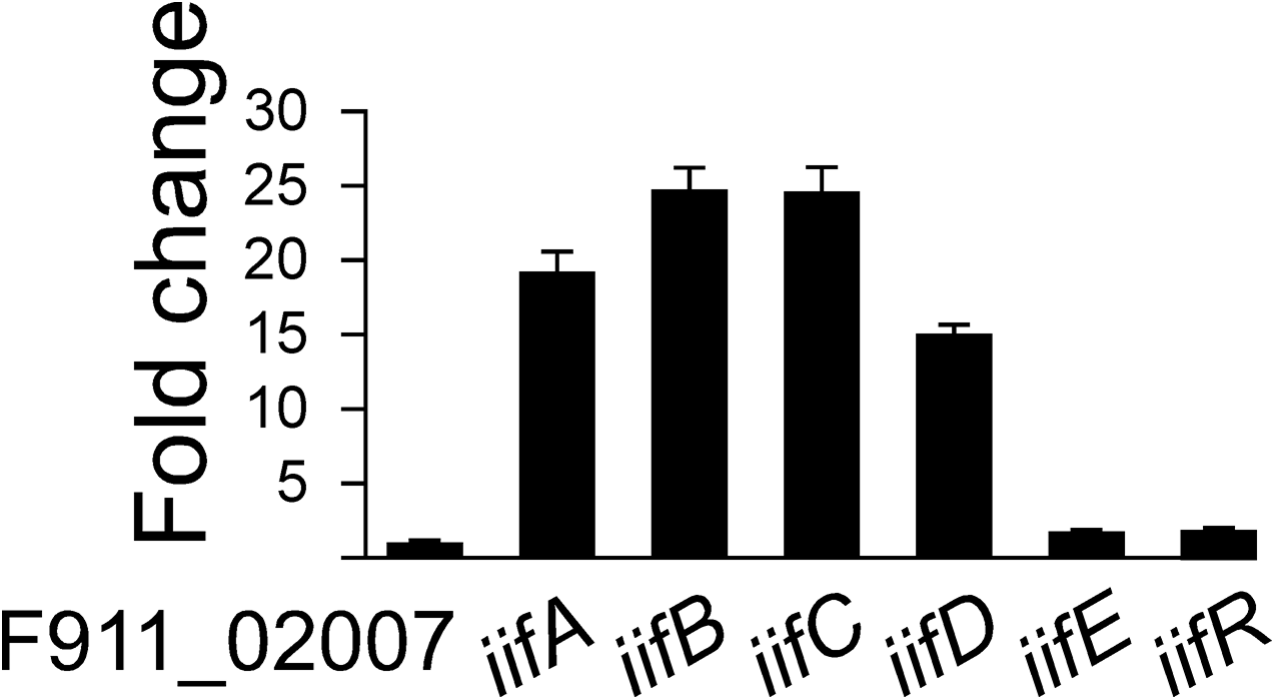
Expression of *iif* genes *A*. *baumannii* ATCC 19606 following the addition of indole. Expression of the *iif* genes was examined by RT-qPCR. Graph shows the fold change for each gene following the addition of 1.5 mM indole after normalization to *gyrB*. Reactions were done in triplicate.

In order to determine the genes functioning in the conversion of indole to indigo in *A*. *baumannii*, RT-qPCR was performed to investigated the differential gene expression of *iifABCDE*, *iifR*, and the gene located immediately upstream of *iifA* (locus_tag F911_02007) in the presence and absence of indole. The expression of *iifA*, *iifB*, *iifC*, and *iifD* was increased in the presence of indole (18.6 ± 1.6 fold, 24.7 ± 1.8 fold, 24.2 ± 1.5 fold, and 14.7 ± 0.6, respectively) (Fig 3). F911_02007, *iifE*, and *iifR*, on the other hand, showed only slight changes in expression (0.8 ± 0.1 fold, 1.5 ± 0.1 fold, and 1.7 ± 0.3 fold, respectively). Therefore, it is likely that one or more of the genes located in the *iifABCD* region of the operon is responsible for indigo production in this bacterial species.

To further investigate the pigment-producing genes located in the fosmid pOXY, transposon insertion analysis was performed with the EZ-Tn*5* <KAN-2> transposon. Transposon tagged fosmids were transformed into *E*. *coli* Epi300. Nine transposon-tagged fosmids, which could not produce blue pigment when expressed in *E*. *coli* were isolated. Sequence analysis of these fosmids revealed that the EZ-Tn*5* <KAN-2> insertion sites of all of these clones were located in the *iifC* gene, indicating that this gene is likely responsible for producing blue pigment when expressed in *E*. *coli*. The EZ-Tn*5* <KAN-2> target sites are listed in S1 Table.

Additionally, a DNA fragment containing the intact *iifC* gene as well as the 223 bp upstream sequence was amplified and subcloned to generate the pSCB-OXY plasmid (also featured in Fig 2). Both the pOXY and pSCB-OXY plasmids were observed to form blue colonies when expressed in *E*. *coli* on LB agar plates.

An IifC expression plasmid (pQE80L-IifC) was constructed for protein overexpression and introduced into two *E*. *coli* strains: DH5α and CY15000 (a *tnaA* mutant). Following expression plasmid transformation, but prior to the addition of IPTG and indoline, overnight cultures of both strains were extracted with ethyl acetate, concentrated, and analyzed by TLC. Unlike *E*. *coli* DH5α(pQE80L-IifC), which produced indigo, the *tnaA* mutant *E*. *coli* CY15000(pQE80L-IifC) was not able to produce indigo or indirubin (S6 Fig). Taken together, these data demonstrate that in *E*. *coli* strains with fully functioning tryptophanase proteins, which will naturally be producing low levels of indole, the expression of *iifC* is sufficient for indigo production. Moreover, in the *E*. *coli* CY15000(pQE80L-IifC) cultures, when the non-functional tryptophanase was bypassed by adding indoline, the culture medium turned a deep pink color when were cultured overnight (S7 Fig).

### Purification of IifC protein and enzyme assay

**Fig 4:**
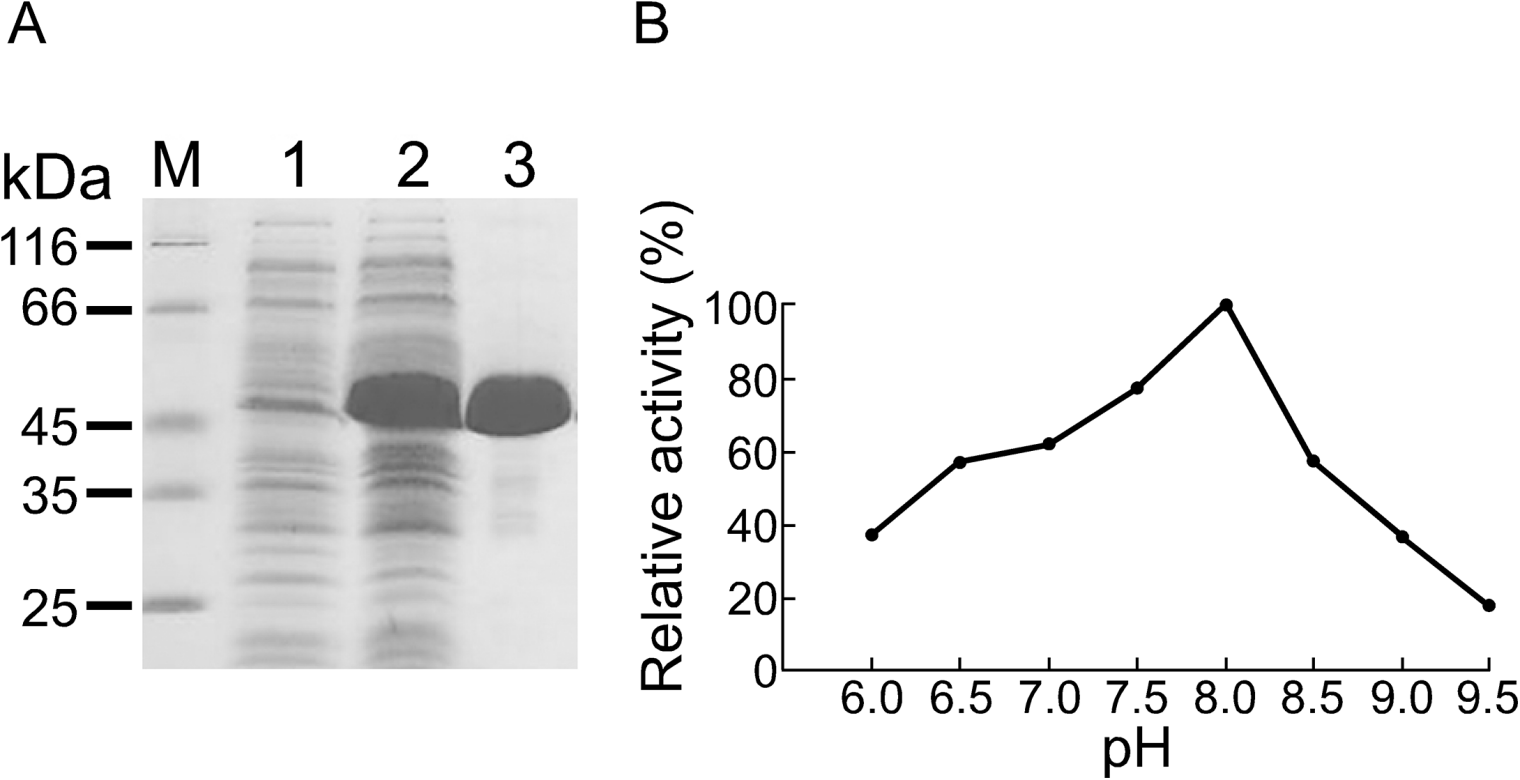
IifC protein purification and kinetics. (A) Overexpression and purification of recombinant IifC. Cell extracts were prepared before (lane 1) and after (lane 2) IPTG induction. Recombinant IifC protein (lane 3) was further purified using Q-Sepharose High Performance anion-exchange column. (B) Enzymatic activity of IifC in the presence of indole in various pH buffers. pH dependent activity assay was carried out in 100 mM potassium phosphate buffer between pH 6.0 and 7.5, 100 mM Tris-HCl buffer between pH 8.0 and 9.5.

IifC protein expression was initially carried out at 37°C with vigorous shaking, resulting in the proteins being well-expressed in soluble form. After sonication to disrupt the cells and centrifugation to remove cell debris, the clarified protein solution (crude extract) was loaded directly onto a 2.6 × 40 cm column of anion-exchange matrix equilibrated with 10 mM phosphate buffer, pH 7.0, containing 5 mM EDTA. Protein was then eluted with a linear, ascending gradient of potassium chloride. The protein was concentrated and observed to be nearly homogeneous (Fig 4A). Further, the color of the purified protein solution was light yellow. FAD content assay indicated that 1 mol of recombinant IifC contained 0.48 mol of FAD. Purification of the IifC protein was 17.6-fold with a yield of 45% (S2 Table), and the optimum pH for the enzyme was observed in the alkaline range (varying between 6.0 and 9.5). The maximum activity rate was achieved at pH 8.0 (Fig 4B). Therefore, all further substrate specificity and kinetic experiments were conducted at pH 8.0.

Next, we investigated the enzyme kinetics of IifC. The conversion of indole into indigo was dependent upon the presence of IifC, NADH, FAD, and indole in the assay. The IifC *K*_*m*_ values for indole and NADH were 0.20 ± 0.05 mM and 0.27 ± 0.03 mM, respectively, while the *k*_*cat*_ was 0.38 ± 0.03 min^-1^.

### Indole toxicity and expression of IifC in the *iifC* and *iifR* mutants

In contrast to wild-type *A*. *baumannii* ATCC19606 (S2 Fig), neither the Δ*iifC* or Δ*iifR* strain could produce indigo when cultured in M9 medium with 3 mM indole. To verify the functions of the *iifC* and *iifR* genes, we performed complementation experiments. Using electroporation, we transformed pComIifC and pComIifR plasmids into Δ*iifC* and Δ*iifR* strains, respectively. Δ*iifC*(pComIifC) and Δ*iifR*(pComIifR) could both produce indigo when cultured in M9 medium with indole (S2 Fig).

**Fig 5:**
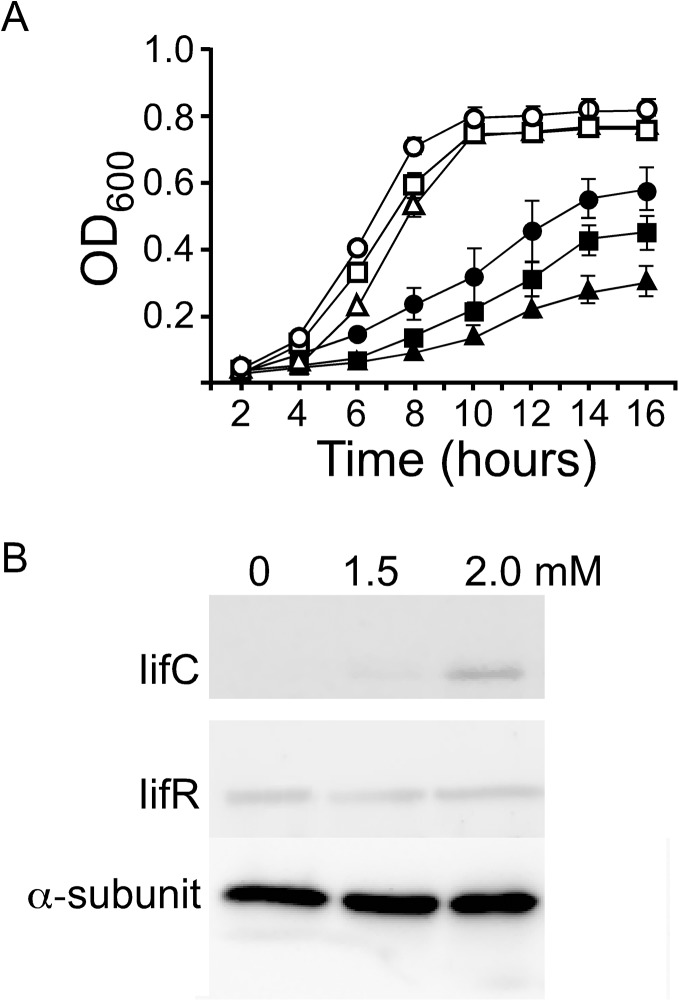
Effect of indole on the growth of wild-type *A*. *baumannii* ATCC 19606 and mutant strains. (A) Wild type (●), *iifC* (■), and *iifR* (▲) mutants were grown in M9 medium containing 0 or 3 mM indole (highlighted by the empty shapes or solid shapes, respectively). The results present the mean ± SD of three independent cultures. The growth of wild-type, Δ*iifC*(pComIifC) and Δ*iifR*(pComIifR) strains is similar. (B) Western blot of the total proteins isolated from *A*. *baumannii* ATCC 19606 cultured in M9 medium with 0, 1.5, and 2.0 mM indole. The alpha-subunit of RNA polymerase (α-subunit) was used as a loading control.

We also compared the growth of wild-type *A*. *baumannii* ATCC19606, Δ*iifC*, Δ*iifR*, Δ*iifC*(pComIifC), and Δ*iifR*(pComIifR) strains cultured in M9 medium with 3 mM indole. From the OD curves, it is evident that, in the absence of indole, the growth of these strains is similar (Fig 5A). However, in the presence of 3 mM indole, the wild-type, Δ*iifC*(pComIifC) and Δ*iifR*(pComIifR) strains grew much more slowly. Further, the growth of the Δ*iifC* and Δ*iifR* strains was further inhibited compared to the wild-type, with the Δ*iifR* strain being more severely affected by indole than the Δ*iifC* strain (Fig 5A).

To further determine the function of IifC in these mutant strains, bacteria were cultured in M9 medium with and without indole, and IifC protein was detected by Western blotting. Our data indicate that IifC protein expression is induced by indole in a dose-dependent manner (Fig 5B). While the levels of IifC increase in *A*. *baumannii* ATCC19606, this strain expressed the same amount of IifR protein when cultured in M9 medium, regardless of the level of indole. Moreover, the *iifR* mutant does not appear to express IifC, irrespective of the presence of indole. These data indicate that while IifC appears to be the main enzyme functioning in converting indole to indigo, IifR likely plays an important regulatory role in this process.

## Discussion

In this study, we have shown that *A*. *baumannii* utilizes the indole-converting protein IifC to protect itself from the effects of this compound at low concentrations, while at higher (3.5 mM) concentrations the indole toxicity overcomes these defenses and inhibits growth. Further, it appears that IifR also plays a regulatory role in this pathway. To our knowledge, this is the first time IifC has been documented to function as an enzymatic defense against indole toxicity in this bacterial strain.

Protection against indole-induced cytotoxicity has been observed in a number of bacterial species. While some species degrade indole or use it as a carbon source [26, 37–39], many species utilize oxidative enzymes that convert indole to non-toxic indigo. Further, recombinant *E*. *coli* expressing exogenous genes from several species, including *Acinetobacter* spp. [21, 23], *Bacillus subtilis* WU-S2B [40], *Burkholderia cepacia* [41], *Pseudomonas* spp. [42–44], *Methylophaga* sp. [45], *Ralstonia eutropha* [46], *Rhodococcus* spp. [47–49], or the bacterial metagenome [50–52] also convert indole to various indigoids. Moreover, in the presence of an aromatic inducer, *Acinetobacter* spp. [21, 22], *Comamonas* sp. [53], and *Pseudomonase* spp. [54–59] also transform indole into indigoids.

*A*. *baumannii* is a notable opportunistic pathogen of the genus *Acinetobacter* [60] that is known to survive in diverse environments, including soil [25]. Further, different aromatic compounds released from plant and crude oil are also present in the soil [61] and *Acinetobacter* spp. can degrade and utilize a variety of these molecules as carbon and energy sources [61]. For example, previous studies have shown that *A*. *baumannii* can use the aromatic compounds benzoate, *p*-hydroxybenzoate and indole-3-acetic acid (IAA) [23, 62]. In our previous report [23] and the present study, we have demonstrated that, like other aromatic compound-degrading bacteria, *A*. *baumannii* can also convert toxic indole to non-toxic indigo. Although these bacteria can oxidize indole, no work has been published investigating the mechanism of this phenomenon.

In the search to understand indole conversion, a number of genes have been discovered that can oxidize this compound to indigo. For example, the *iacA* gene of *A*. *baumannii*, which encodes an indole oxygenase, can be induced by the plant hormone IAA. However, bacteria harboring an *iacA* mutation can still convert indole into indigo, indicating that there is likely another indole oxygenase gene in *A*. *baumannii* that is the primary enzyme in this reaction.

Thus, to investigate other genes involved in indigo production, we isolated a fosmid clone, pOXY, from our *A*. *baumannii* genomic library. Sequence analysis revealed that *A*. *baumannii* harbors the *iif* operon, a sequence of five structural genes that is regulated by an AraC/XylS family member. Interestingly, it appears that the *iif* operon is only present in two bacterial species, *A*. *baumannii* and *P*. *syringae* pv. *actinidiae*. While these genes in both species are organized identically and display strong similarity, the average G + C content of the *iif* operon is quite different, indicating that it has existed in these two organisms for a long time. Unfortunately, aside from *iifC*, which was characterized in this study, the functions of the rest of the *iif* genes are still unclear. For example, sequence analysis showed that the *iifA* gene encodes a dienelactone hydrolase-like protein. Dienelactone hydrolases are involved in the degradation of 2, 4-dichlorophenoxyacetic acid (2, 4-D) and other chloroaromatics [63], but the function of IifA, and how *A*. *baumannii* degrades 2, 4-D is still unknown.

Notably, the third gene of the *iif* operon, *iifC*, appears to be the primary gene involved in indigo production and indole resistance. Indigo and indigoids are both important dye and drug compounds that are produced by chemical synthesis, isolated from plant sources, and synthesized by bacteria. Interestingly, because *E*. *coli* naturally produces indole, recombinant *E*. *coli* engineered to also express exogenous indigo-producing genes (IPGs) will be blue in color because the IPG will convert the endogenous indole to indigo. Therefore, recombinant *E*. *coli* can be exploited as a host for gene cloning and IPG isolation and analysis.

Since their initial discovery in 1983, many IPGs have been identified, including the cytochrome P450 enzymes and a variety of flavoproteins. Aside from the genes encoding the cytochrome P450 enzymes, these IPG encoded flavoproteins can be roughly divided into two types: Type I, which contain an FAD-binding domain (FBD); and Type II, which do not contain this domain. Table 3 lists the Type I flavoproteins that can oxidize indole in *E*. *coli*. We have previously showed that the Type II IPGs belong to the acyl-CoA dehydrogenase family [23]. Notably, the acyl-CoA dehydrogenase-like flavoproteins encoded by these IPGs do not have a conserved FBD, but contain the Pfam domains Acyl-CoA-dh-N and Acyl-CoA-dh-2 or Acyl-CoA-dh-N and Acyl-CoA-dh-M. Further, these enzymes are all similar in size, ranging from 387 to 416 amino acids [23]. On the other hand, the sizes of the flavoproteins encoded by the Type I IPGs are larger than 400 amino acids (Table 3). Moreover, the protein domains of these flavoproteins are much more diverse that those of Type II IPGs and can contain flavoprotein monooxygenase (FMO)-like, FAD_binding_3, or Pyr_redox_2 Pfam domains. *iifC* is a Type I IPG, but after a Pfam database search, no significant domains were identified. However, similar to styrene monooxygenase, IifC also appears to contain the FAD-binding fingerprints GxGxxG, GG, and Dx6G at amino acid positions 8, 119, and 140, respectively.

**Table 3:** Indigo producing genes encoding FAD containing proteins.

Gene/Source	Length (bp)	Product size (aa)	Protein Accession No.	FAD-binding sequence (GxGxxG-GG-DxxxxxxG)	FAD/NAD(P) interaction motif (GDxxxxxxP)	Pfam family^a^	Reference	Pfam family
*iifC/A*. *baumannii*	1239	412	ENW75087	GAGQSG-GG-ELVLLAAG	ADALVVNDP	No significant matches	This study	No significant matches
*fmo*/*Methylophaga* sp.	1371	456	AAM18566	GAGPSG-//-DYVVCCTG	RDVIMGRLP	FMO-like	[45]	FMO-like
*smo*/metagenome	1251	416	ABV24041	GAGIAG-GG-DLLVVSSG	GDVHSVVDP	FAD_binding_3	[52]	FAD_binding_3
*styA2B*/*R*. *opacus* 1CP	1221	406	ACR43973	GAGQAG-GG-DLVILAAG	ADVVVLNDP	Pyr_redox_2	[49]	Pyr_redox_2

^a^Determined by searching the Pfam database.

In addition to flavins, the cofactor NAD(P)H is also involved in FMO reactions. Flavins are reduced by NAD(P)H via electron transfer and then sequentially converted to hyroperoxyflavins, which are an unstable intermediate. FMOs can bind and stabilize these hyroperoxyflavins, and subsequently oxygenate a variety of substrates [64]. In most two-component FMOs, a reductase gene is located immediately downstream of a monooxygenase gene [52]. For example, *C2-hpah* and *C1-hpah* encode a *p*-hydroxyphenylacetate hydroxylase, a two-component FMO of *A*. *baumannii* isolated from Thai soil [65]. Further, in *P*. *fluorescens* ST *styA* and *styB* genes encode a two-component styrene monooxygenase (SMO) [66]. Notably, the Gdx6P motif, important for the FAD/NAD(P) interaction [49], is also found at amino acid position 292 of IifC. In addition, our results also shown that 1 mol of purified IifC contained 0.48 mol of FAD. Due to the dissociation of non-covalently-bound FAD from proteins during purification, both FMO [45] and IifC purified from *E*. *coli* contained approximately 0.5 mol of FAD per mol of these two proteins. Although StyA2B contains the FAD-binding fingerprints, recombinant His_10_-StyA2B purified from *E*. *coli* with Ni-chelate affinity chromatography contained trace amounts of FAD [49]. Therefore, the binding of FAD to FAD-binding protein in the buffer for purification may have influenced the dissociation of FAD from enzymes. Further, we also discovered that *iifD*, located directly downstream of *iifC*, is a putative flavin oxidoreductase, which could be functioning as one component of the two-component FMO. Like C2-hpah and StyA, IifC is also larger than IifD. Taken together, these data suggest that the proteins encoded by the *iifC* and *iifD* genes could constitute a putative two-component FMO.

Like Fmo protein of *Methylophage* sp. [45], recombinant IifC protein purified from *E*. *coli* binds to FAD. As a flavoprotein oxygenase, the purified IifC contains FAD, preventing the determination of the *K*_*m*_ value for this cofactor. However, for the other cofactors, indole and NADH, IifC protein has a *K*_*m*_ value of 0.20 ± 0.05 mM and 0.27 ± 0.03 mM, respectively, and a *k*_*cat*_ of 0.38 ± 0.03 min^-1^. Notably, the *K*_*m*_ for indole is much lower than the concentration required to significantly inhibit the growth of cultured *A*. *baumannii*. Further, the indole *K*_*m*_ of IifC was four times lower than that previously found for IacA (0.80 ± 0.04 mM) and the catalytic efficiency of the enzyme (*k*_*cat*_/*K*_*m*_) of IifC (1.9 min^-1^mM^-1^) was higher than that of IacA (1.1 min^-1^mM^-1^) [23]. Thus, it would appear that IifC is more efficient at converting indole to indigo. This was not surprising as we suspect indole may be the natural substrate of IifC, while the natural substrate of IacA is IAA. However, because IAA and indole are structurally similar, IacA could also oxidize indole [23], just at a lower efficiency. In fact, when examining current research it appears that although other enzymes encoded by IPGs can oxidize indole, the natural substrate of these enzymes is not indole. For example, cytochrome P450 enzymes, which have wide substrate specificity [67], can oxidize indole. In addition, O’Connor et al. [56] reported that since indole and styrene are very similar structurally, SMOs can also use indole even though this is not their natural substrate. However, while these other IPGs may have the ability to convert indole, IifC is induced by indole *in vivo*, supporting our hypothesis that indole is the natural substrate of this enzyme, making it the key player in indole resistance. Not surprisingly, indoline, a compound that is structurally similar to indole, was also converted to an uncharacterized water-soluble pigment in a similar manner by IifC.

While IifC is likely the primary enzyme involved in indole conversion, mutagenesis and complementation analysis showed that IifR is required for IifC expression. Sequence analysis indicates that IifR may be a member of the AraC/XylS family, all of which, with the exception of CelD, are positive transcriptional activators [36, 68]. Notably, AraC/XylS family members are known to regulate bacterial genes involved in carbon metabolism, stress response, and manifestation of pathogenesis [36, 69]. Therefore, in the presence of the appropriate inducer, AraC/XylS-like proteins could likely activate cognate operon expression. In fact, the expression of several operons involved in the degradation of aromatic compounds has been shown to vary depending on the concentration of the AraC/XylS inducer [70]. Interestingly, in the present study, we observed that the expression of IifC was activated by indole in a dose-dependent manner. Taken together, these results indicate that IifR is an AraC-like protein that functions to control *iif* operon expression. However, while the N-terminal non-conserved domain of AraC and XylS can bind arabinose and benzoate [71, 72], respectively, further study is needed to determine where IifR can bind indole. Alternatively, another activator/cascade could be modulating this regulatory protein, such as that observed for other AraC/XylS family members [70, 72]. Western blot analysis also revealed that the expression of IifR was constitutive irrespective of indole concentrations, indicating that this protein may not be regulating the indole-induced stress response alone.

Notably, a number of other indole-inducible proteins involved in indole resistance have been characterized previously, in addition to IifC and IifR. For example, AhpC, found in *E*. *coli*, and a novel 36-kDa protein found in *Brevibacterium flavum* have both been reported to be induced by indole [9]. We can also not rule out the possibility that IifR could be an activator for any number of other indole-inducible genes in this pathway. Further, we suspect that these various other genes and regulatory proteins may also be contributing synergistically with IifC and IifR to help protect the bacteria from the toxic effects of indole and, therefore, warrant further investigation.

In conclusion, our work suggests that indole oxidization (detoxification) in *A*. *baumannii* is performed by IifC, which is in turn controlled by a member of the AraC/XylS family, IifR. While these data help advance our understanding of how bacteria counteract the toxicity of the aromatic compound, indole, additional work is necessary to elucidate the full signaling cascade and functional mechanism underlying this phenomenon.

## Supporting Information

S1 Fig The effect of indole and indigo on the growth of *A*. *baumannii*. *A*. *baumannii* ATCC 19606 was grown in M9 medium containing 0.1% ethanol as carbon source (control). To test the toxicity of indole and indgo, *A*. *baumannii* ATCC 19606 was cultivated in M9 medium supplemented with 5 mM indole (indole) or 5 mM indigo (indigo). After incubation for 16 h at 37°C, the cell cultures were serially diluted and 10 μl-drops of selected dilutions were plated onto LB agar plates for viable counts.
(DOCX)

S2 Fig *iifC* and *iifR* involved in indigo production of *A*. *baumannii* ATCC 19606. Indigo was produced by wild-type *A*. *baumannii* ATCC 19606 (A), Δ*iifC*(pComIifC) (B), and Δ*iifR*(pComIifR) (C). In contrast, Δ*iifC* (D) and Δ*iifR* (E) cannot produce indigo. These strains were grown in LB broth overnight. Bacteria from 0.5 ml of the overnight cultures was collected by centrifugation, washed with M9 medium, and transferred to fresh M9 medium (50 ml) supplemented with 3.0 mM indole and 0.1% ethanol. Indigo was observed in the medium after 16 h.
(DOCX)

S3 Fig Examination of *iifABCDE* polycistronic mRNA by reverse transcription PCR (RT-PCR). (A) Diagram of the intergenic regions of *iifA-iifB*, *iifB-iifC*, *iifC-iifD*, and *iifD-iifE* (represented by thick black lines) that were amplified by RT-PCR. (B) The RT-PCR amplification products were analyzed by agarose gel electrophoresis. RT-PCR amplifications were conducted with reverse transcriptase (lanes 1–4) or without reverse transcriptase (negative control; lanes 5–8) using total RNA as the template. Lanes 1, 2, 3, and 4 are the RT-PCR products of the intergenic region of *iifAB*, *iifBC*, *iifCD*, and *iifDE*, respectively. These cDNA were not amplified in the negative control lanes. Lane M: 100-bp ladder marker.
(DOCX)

S4 Fig Amino acid alignment of IifC and styrene monooxygenase. Asterisks indicate identical amino acids in IifC, StyA, and StyA2B. The FAD-binding fingerprints GxGxxG, GG, and Dx6G are also shown. IifC showed 64.8% (268/413), 59.8% (241/403), and 47.4% (196/413) identity to MoxY (uncultured bacterium; GenBanK accession No. ABQ12175), StyA1 of *Rhodococcus opacus* 1CP (GenBanK accession Nos. ACR43973 and AFO70154) and StyA2B of *R*. *opacus* 1CP (GenBanK accession Nos. ACR43974 and AFO70155), respectively.
(PDF)

S5 Fig Amino acid alignment of IifR and two defined members of the AraC/XylS family. Multi sequence alignment of the DNA binding domains of the IifR of *A*. *baumannii* (IifR/Ab), IifR of *P*. *syringae* pv. *actinidiae* (IifR/Ps), AraC of *E*. *coli* (AraC/Ec; SWISSPROT accession no. P03021), and XylS of *P*. *putida* (XylS/Pp; SWISSPROT accession no. P07859) were carried out manually. This region contains two potential helix-turn-helix (HTH) DNA binding motifs (first and second HTH motifs). Underlined sequences indicate the helical regions.
(PDF)

S6 Fig Indigoid production by recombinant *E*. *coli* carrying the *iifC* gene. Ethyl acetate extracts of *E*. *coli* DH5α(pQE80L-OXY) (lane 2) and *E*. *coli* CY15000(pQE80L-OXY) (lane 3) culture broth were analyzed by TLC. Indirubin (lane 1) and indigo (lane 4) were used as marker.
(DOCX)

S7 Fig Oxidation of indoline by IifC. *E*. *coli* CY15000(pQE80L) (tubes 1–3) and *E*. *coli* CY15000(pQE80L-OXY) (tubes 4–6) were inoculated into 3 ml of LB broth containing 1 μl (tubes 1 and 4), 2 μl (tubes 2 and 5), or 3 μl (tubes 3 and 6) of indoline. After incubating for an additional 16 h, the medium turned a deep pink color in the cultures of *E*. *coli* CY15000(pQE80L-OXY) (tubes 4–6).
(DOCX)

S1 Methods Mutation of the *iifC* and *iifR* genes and complementation testing. (DOCX)

S1 Table The <KAN-2> transposon insertion sites within *iifC*. (DOCX)

S2 Table Purification of recombinant IifC. (DOCX)
