# Supplementary figures and images for: Detoxification of Indole by an Indole-Induced Flavoprotein Oxygenase from Acinetobacter baumannii
Source: PLoS One. 2015 Sep 21;10(9):e0138798. doi: 10.1371/journal.pone.0138798 (PMC4577076; doi:10.1371/journal.pone.0138798)

1. **(B)**


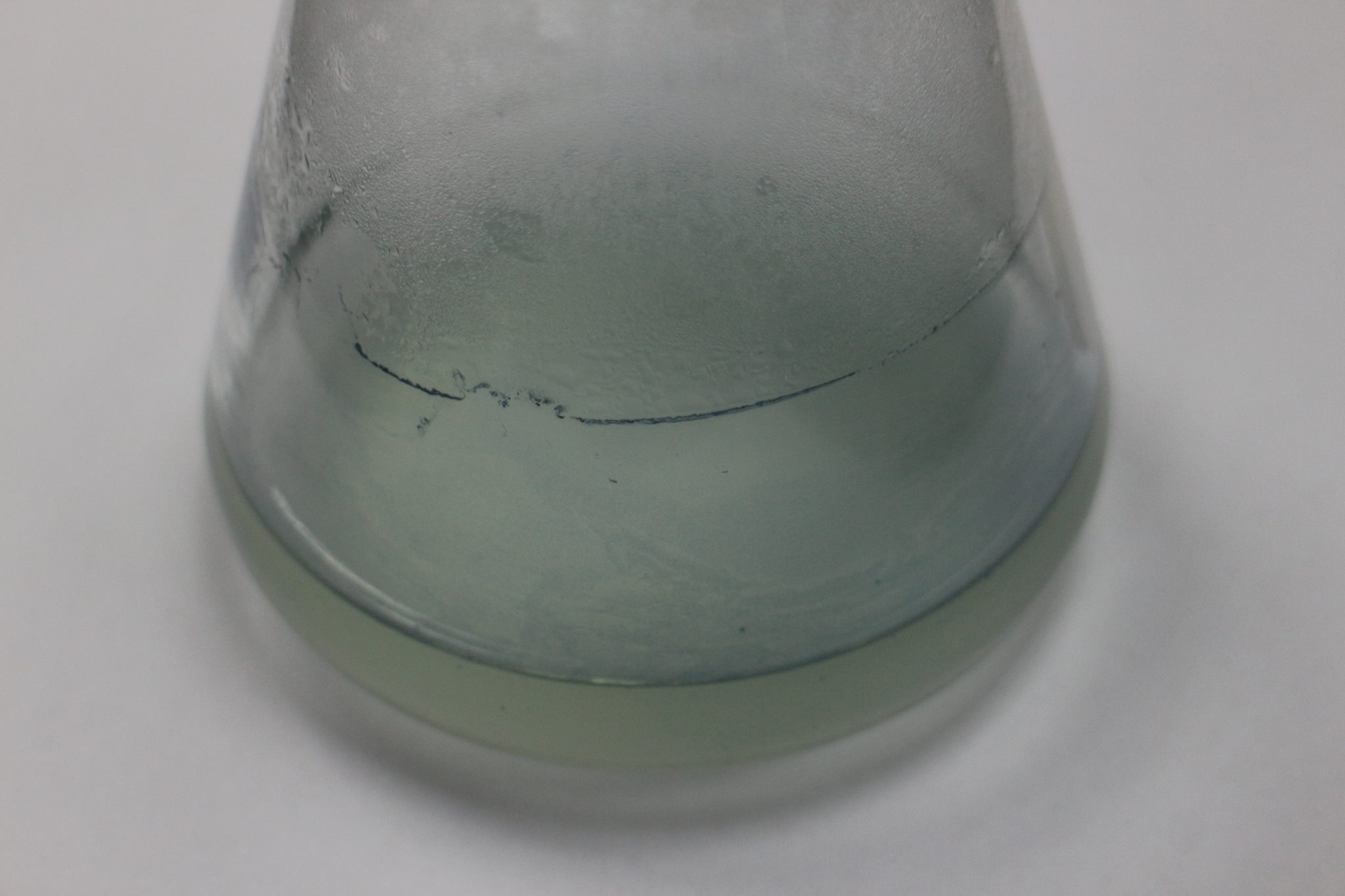

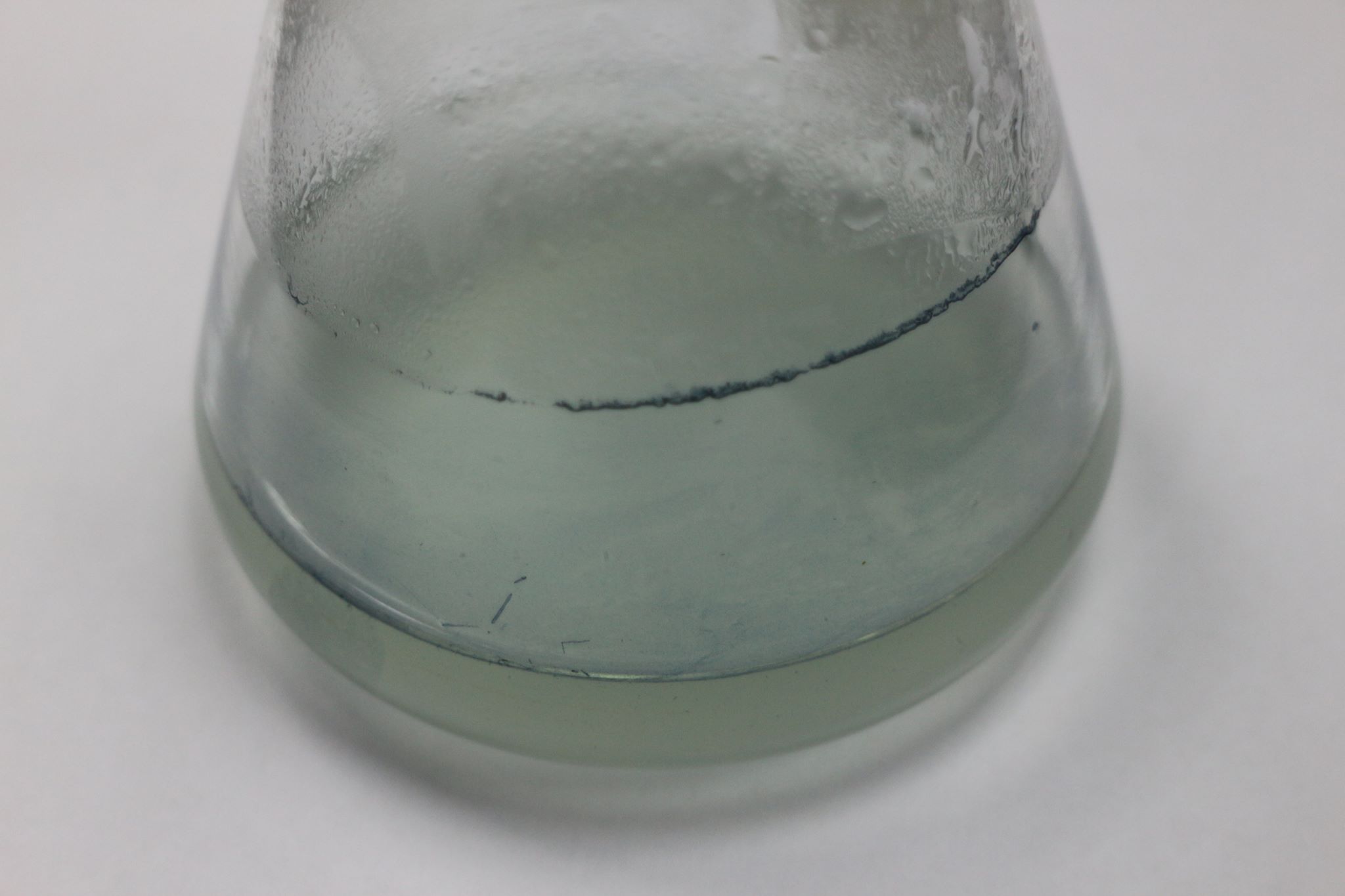


**(C) (D)**


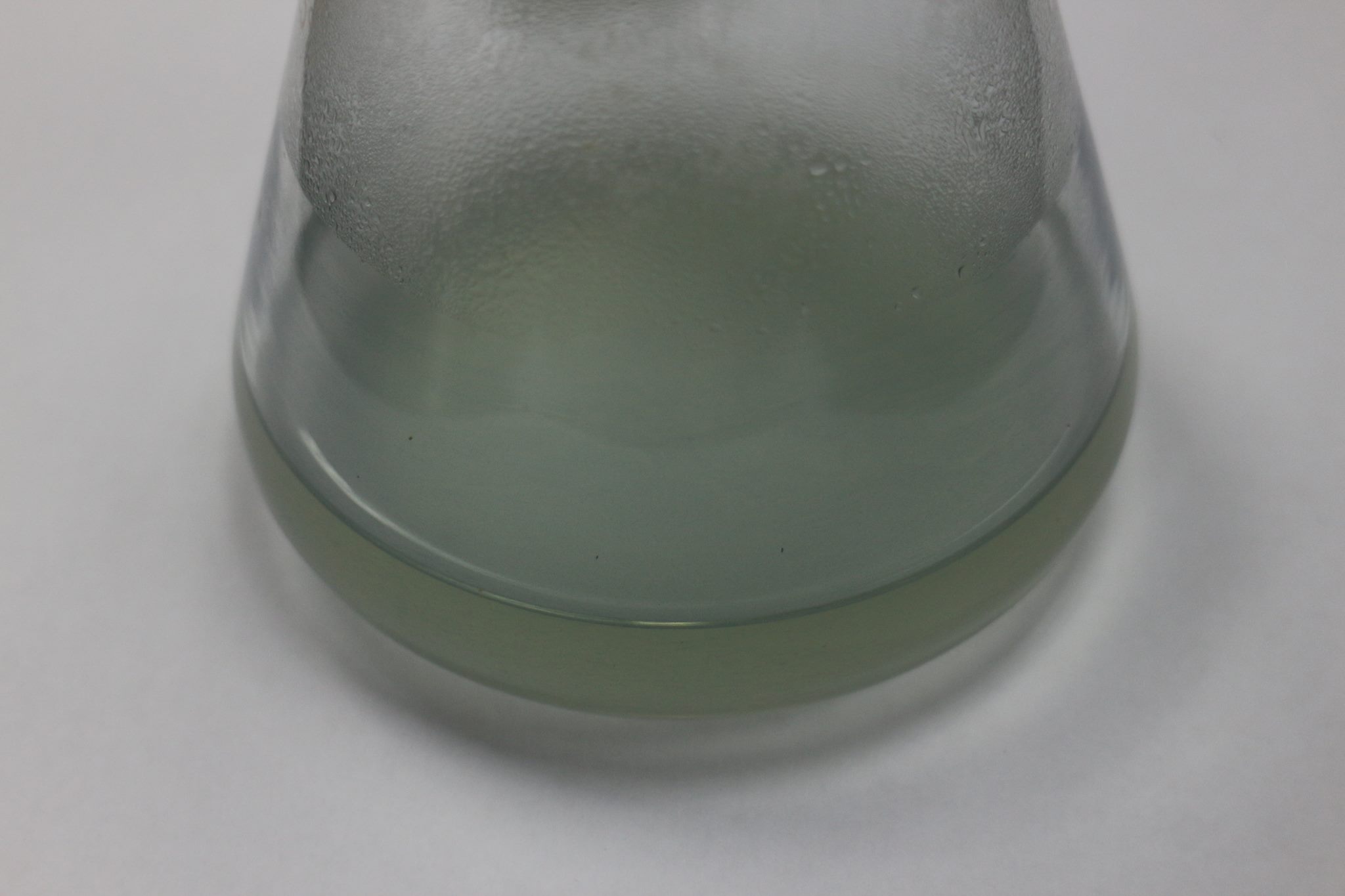

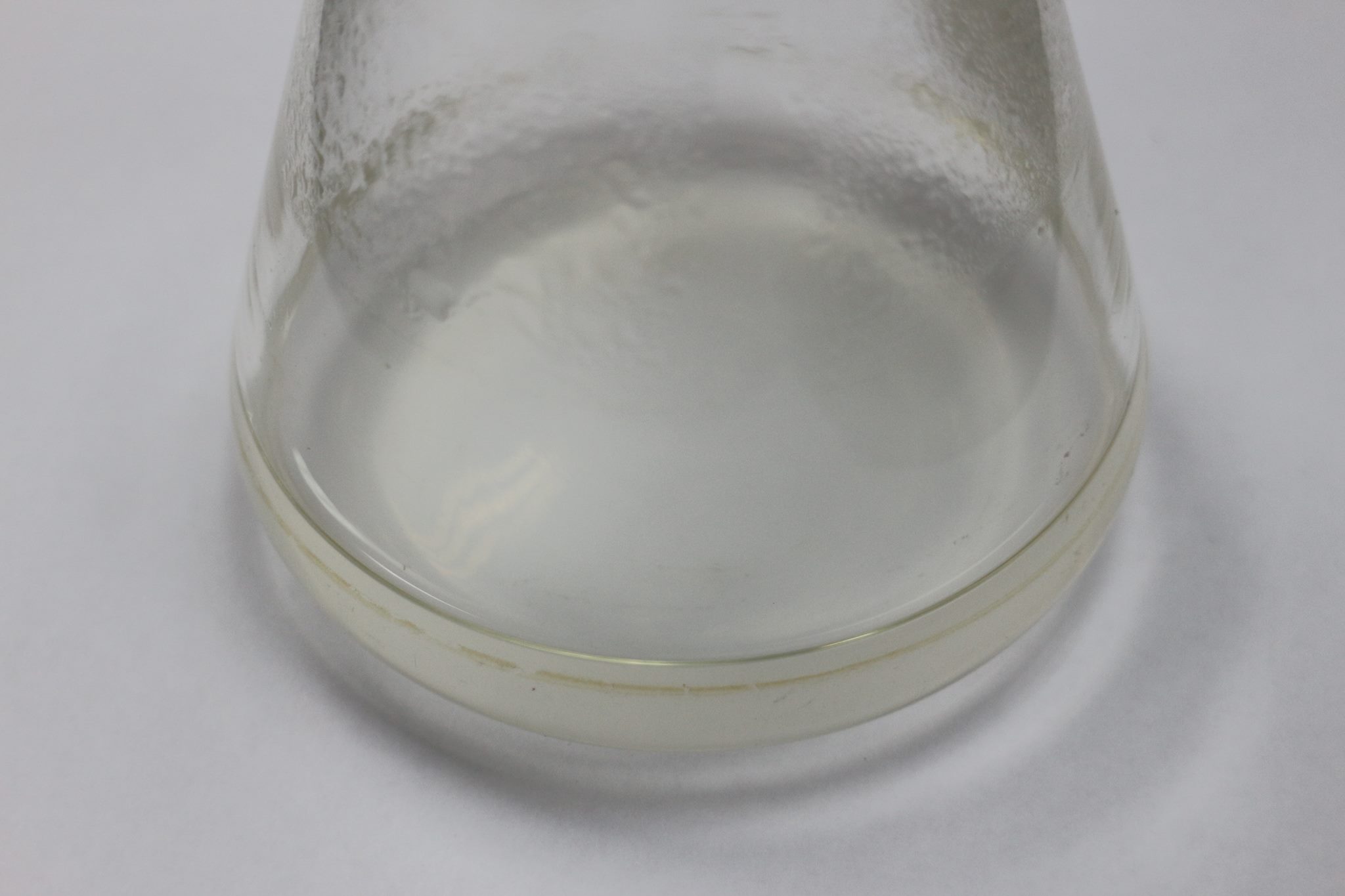


**(E)**


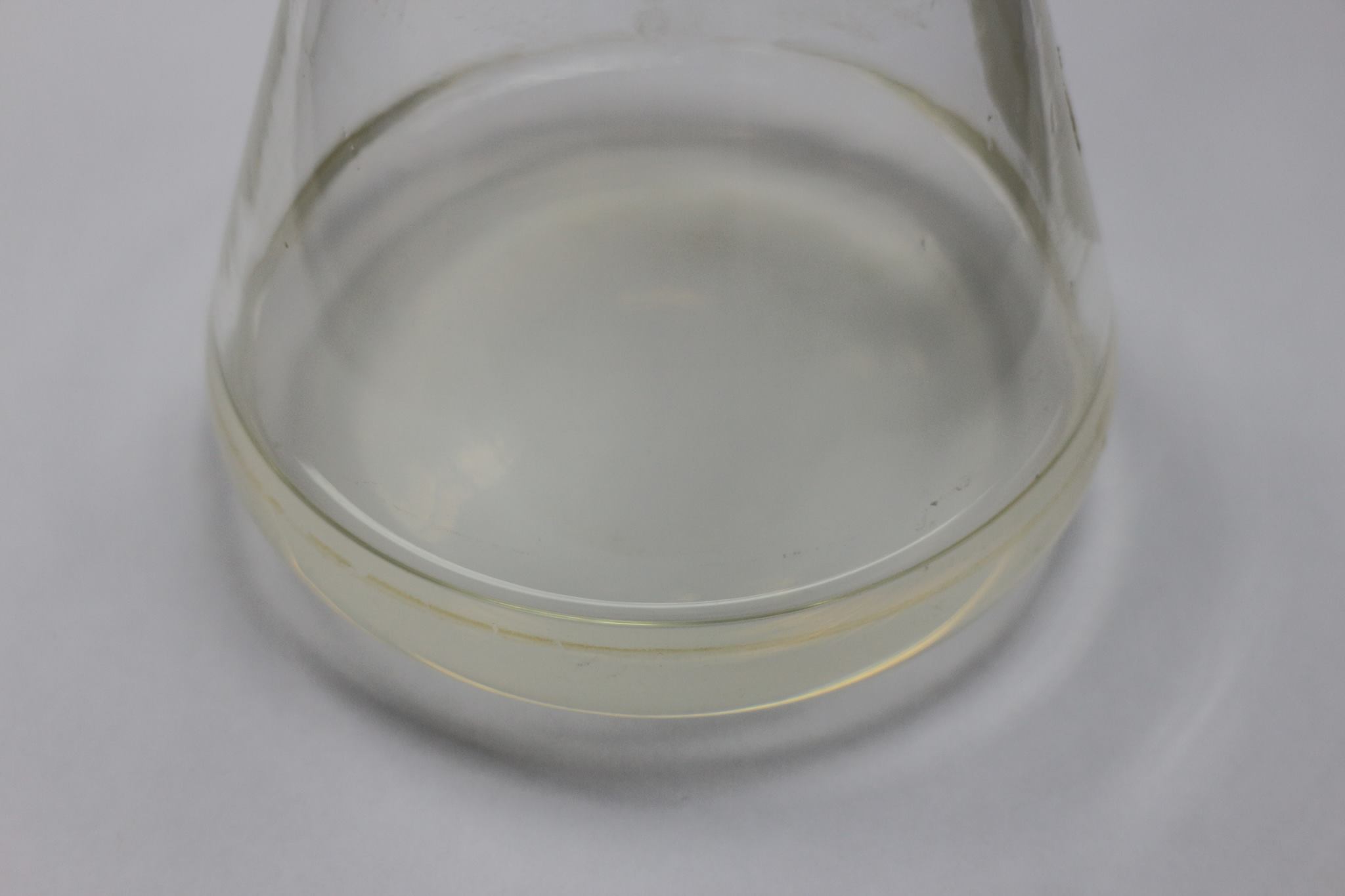

Supplement: S2 Fig — Indigo was produced by wild-type A. baumannii ATCC 19606 (A), ΔiifC(pComIifC) (B), and ΔiifR(pComIifR) (C). In contrast, ΔiifC (D) and ΔiifR (E) cannot produce indigo. These strains were grown in LB broth overnight. Bacteria from 0.5 ml of the overnight cultures was collected by centrifugation, washed with M9 medium, and transferred to fresh M9 medium (50 ml) supplemented with 3.0 mM indole and 0.1% ethanol. Indigo was observed in the medium after 16 h. (DOCX) [file pone.0138798.s002.docx]

**(A)**


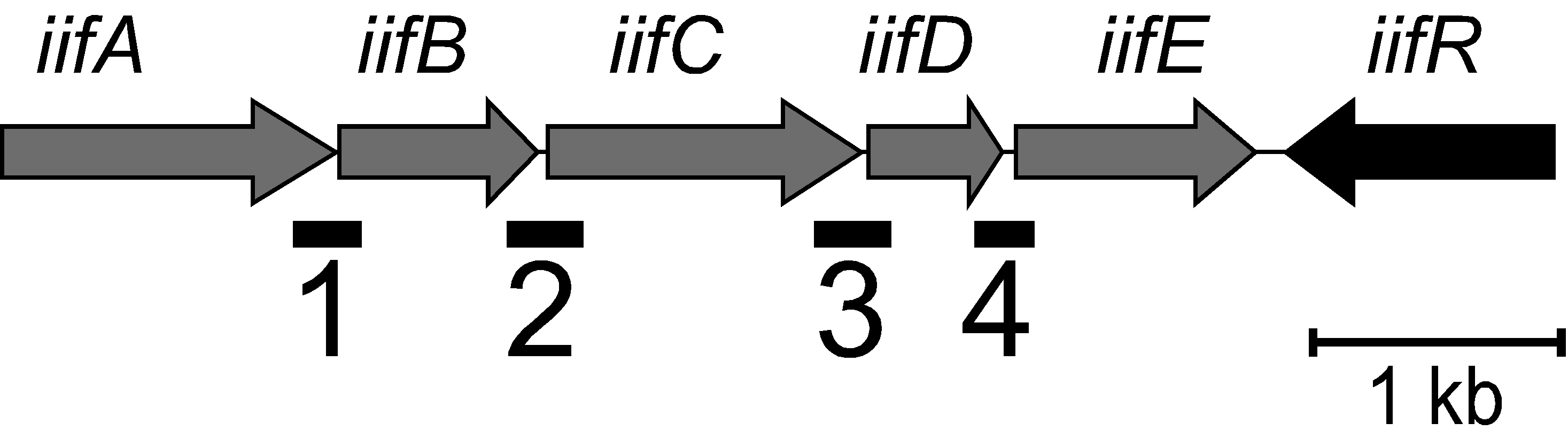


**(B)**


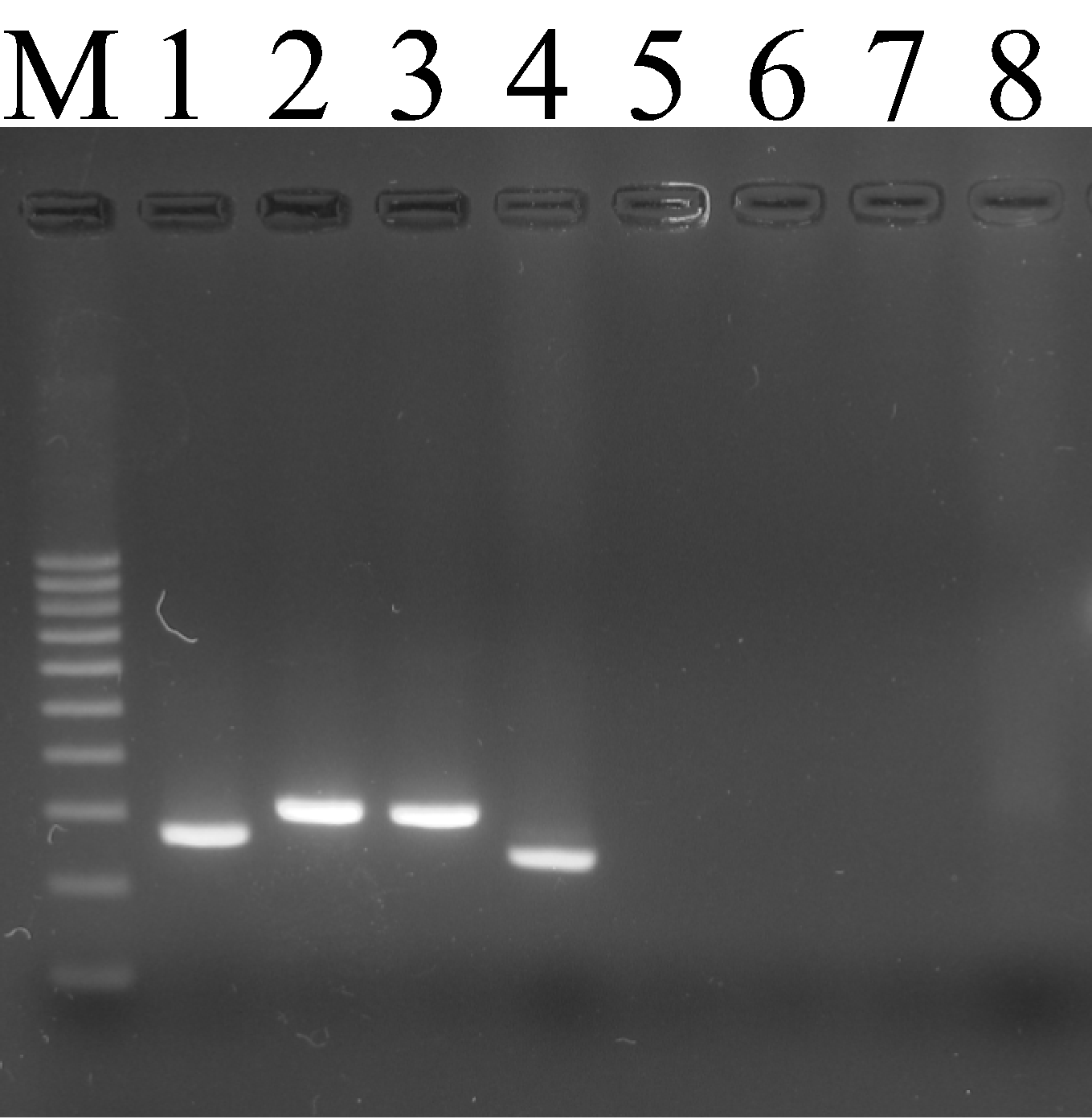

Supplement: S3 Fig — (A) Diagram of the intergenic regions of iifA-iifB, iifB-iifC, iifC-iifD, and iifD-iifE (represented by thick black lines) that were amplified by RT-PCR. (B) The RT-PCR amplification products were analyzed by agarose gel electrophoresis. RT-PCR amplifications were conducted with reverse transcriptase (lanes 1–4) or without reverse transcriptase (negative control; lanes 5–8) using total RNA as the template. Lanes 1, 2, 3, and 4 are the RT-PCR products of the intergenic region of iifAB, iifBC, iifCD, and iifDE, respectively. These cDNA were not amplified in the negative control lanes. Lane M: 100-bp ladder marker. (DOCX) [file pone.0138798.s003.docx]

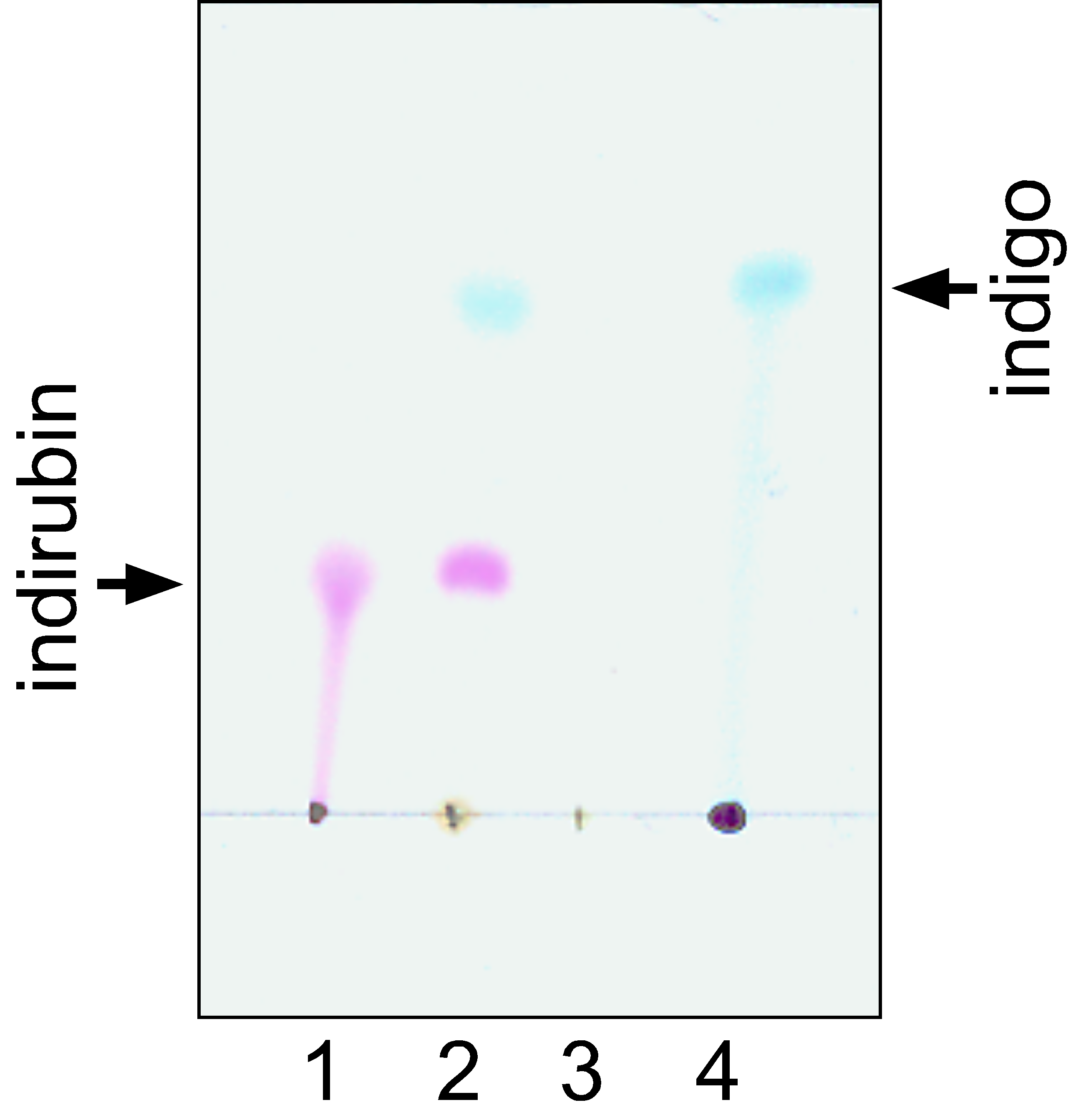

Supplement: S6 Fig — Ethyl acetate extracts of E. coli DH5α(pQE80L-OXY) (lane 2) and E. coli CY15000(pQE80L-OXY) (lane 3) culture broth were analyzed by TLC. Indirubin (lane 1) and indigo (lane 4) were used as marker. (DOCX) [file pone.0138798.s006.docx]

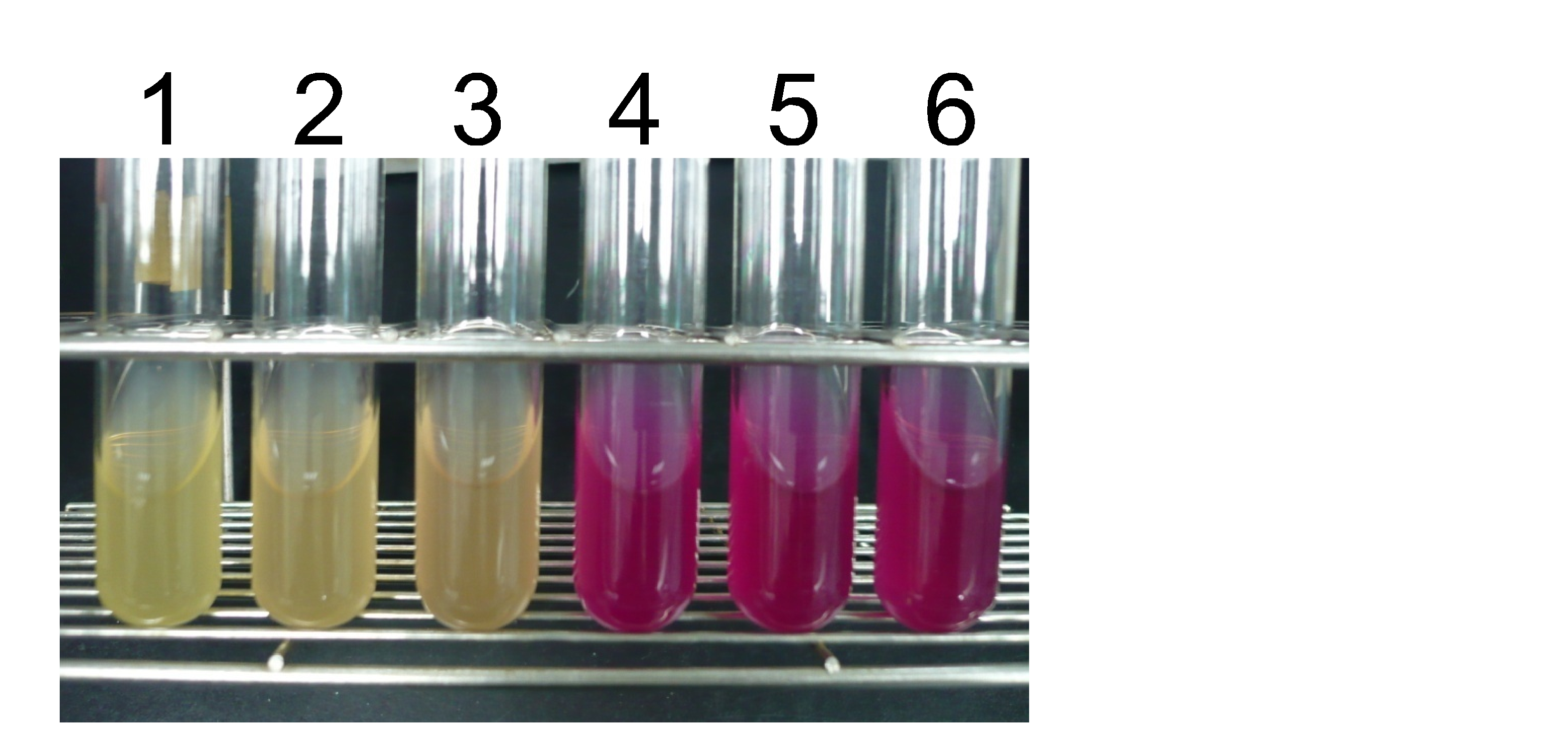

Supplement: S7 Fig — E. coli CY15000(pQE80L) (tubes 1–3) and E. coli CY15000(pQE80L-OXY) (tubes 4–6) were inoculated into 3 ml of LB broth containing 1 μl (tubes 1 and 4), 2 μl (tubes 2 and 5), or 3 μl (tubes 3 and 6) of indoline. After incubating for an additional 16 h, the medium turned a deep pink color in the cultures of E. coli CY15000(pQE80L-OXY) (tubes 4–6). (DOCX) [file pone.0138798.s007.docx]
