# Supplementary material for: Detoxification of Indole by an Indole-Induced Flavoprotein Oxygenase from Acinetobacter baumannii
Source: PLoS One. 2015 Sep 21;10(9):e0138798. doi: 10.1371/journal.pone.0138798 (PMC4577076; doi:10.1371/journal.pone.0138798)
Supplement: S5 Fig — Multi sequence alignment of the DNA binding domains of the IifR of A. baumannii (IifR/Ab), IifR of P. syringae pv. actinidiae (IifR/Ps), AraC of E. coli (AraC/Ec; SWISSPROT accession no. P03021), and XylS of P. putida (XylS/Pp; SWISSPROT accession no. P07859) were carried out manually. This region contains two potential helix-turn-helix (HTH) DNA binding motifs (first and second HTH motifs). Underlined sequences indicate the helical regions. (PDF) [file pone.0138798.s005.pdf]

```
                              First HTH                                                   Second HTH
IifR/Ab  255     VEILAEHAGVSVRTLFTGFKNYLGTTPMSYLKELRFEQAHLELMHNENLS  VTDVAFKWGFTHLGRFSQEYKRRYGELP 332
IifR/Ps  252     IEQLAEHAGVSVRTLFSGFREFRNTSPMAFLRHVRMERVHLELRNPGTDS  VTDIAMKWGFAHLGRFSQEYRKHYGELP 329
AraC/Ec  197     IASVAQHVCLSPSRLSHLFRQQLGISVLSWREDQRISQAKLLLSTTRM   PIATVGRNVGFDDQLYFSRVFKKCTGASP 273
XylS/Pp  230  LERLAELAMMSPRSLYNLFEKHAGTTPKNYIRNRKLESIRACLNDPSANVR   SITEIALDYGFLHLGRFAENYRSAFGELP 309
Consensus sequence   A-----S---L---F----G----------R---A---L--------I--I----GF-----F---FR---G--P
                                                                     V  V     Y          K
```
