# Supplementary material for: Detoxification of Indole by an Indole-Induced Flavoprotein Oxygenase from Acinetobacter baumannii
Source: PLoS One. 2015 Sep 21;10(9):e0138798. doi: 10.1371/journal.pone.0138798 (PMC4577076; doi:10.1371/journal.pone.0138798)
Supplement: S1 Methods — (DOCX) [file pone.0138798.s008.docx]

**S1 Methods**

**Construction of the *iifC* mutant.** Conjugation-mediated allelic exchange was used for the construction of the *iifC* mutant. We started with the construction of the suicide plasmid for allelic exchange. To this end, we performed PCR using the OxyF and OxyR primers, and pOXY-*iifC*::KAN-2 (the transposon insertion site was between nucleotides 400 and 401 downstream of the ATG start site of *iifC*) as the template.

The PCR product was ligated into the plasmid backbone (R6Kγ*oir* and *mob* gene) of pBSL181. The backbone of pBSL181 was then amplified with phosphorylated PCR primers (BslF and BslR) using pBSL181 as the template [1], generating pBiifC:: KAN-2 plasmid. pBiifC:: KAN-2 was transformed into *E. coli* S17-1λ*pir* to produce an *E. coli* donor strain for conjugation with *A. baumannii* ATCC19606.

Five hundred microliters of overnight cultures of E. *coli* S17-1 λ*pir* (pBiifC::KAN-2) and *A. baumannii* ATCC 19606 were mixed and centrifuged at 10,000 × *g* for 5 min. The cell pellet was washed twice with 500 µl conjugation buffer (10 mM MgSO_4_), resuspended in 30 µl conjugation buffer and spread onto a 0.45-µm-pore-size filter (S-PAK membrane; Millipore Inc, Bedford, MA) placed on LB agar. The plate was incubated at 37°C for 6 h. The filter was removed and the transconjugants were selected on LB plates with ampicillin (100 µg/ml), chloramphenicol (12.5 µg/ml), and Kanamycin (40 µg/ml). PCR was carried out with the BslF and BslR primers using the chromosome of the *iifC* mutant as template. The PCR product was sequenced to verify the disruption of the *iifC* gene by the EZ-Tn*5*<KAN-2> transposon.

**Construction of the *iifR* mutant.** The *iifR* mutant was constructed using the same methods described for the *iifC* mutant above. We started with the construction of the suicide plasmid for allelic exchange. We performed PCR using *A. baumannii* ATCC 19606 chromosome DNA as template and the IifRF and IifRR primers to amplify the *iifR* gene. The PCR product was ligated into linearized pJET1.2 vector (Fermentas Thermo Fisher Scientific Inc.; Wilmington, DE) to generate the pJiifR plasmid. *In vitro* transposition was used to insert the EZ-Tn*5*<KAN-2> transposon (Epicentre Illumina; Madison, WI) into pJiacR. Sequencing was the performed and we selected a clone, namely pJacR::KAN-2 with the transposon inserted between nucleotides 186 and 187 downstream of the ATG start site of *iifR*. Next, PCR was performed using pJacR::KAN-2 as the template and IifRF and IifRR as the primers. The PCR product was ligated to R6Kγ*ori* and *mob* gene of pBSL181 to generate the pBiifR::KAN-2 plasmid, which was then transformed into *E. coli* S17-1λ*pir*. Selection was done as is described for the *iifC* mutant above.

**Complementation of the *iifR* mutant.** To amplify the full length *iifR* gene, PCR was performed using *A. baumannii* ATCC 19606 chromosome DNA as the template and the cIifRF and cIifRR primers. cIifRF and cIifRR primers harbor *Bam*HI sites at the 5’end. The PCR product included 487 bp upstream of the *iifR* promoter region. The PCR product was ligated into the pJET1.2 vector (Fermentas) to generate the pJCiifR plasmid. DNA sequencing was used to confirm the sequence of the insert in the pJCiifR plasmid. Next, the BamHI fragment carrying *iifR* promoter region and *iifR* gene from pJCiifR was ligated to *Bam*HI-digested pMMB67EH [2] to generate the complementing plasmid pComIifR. pComIifR was electroporated into the *A. baumannii* *iifR* mutant according to a method described elsewhere [3]. Transformants were selected on LB agar containing ampicillin (500 µg/ml).

**Complementation of the *iifC* mutant.** Complementation of the *iifC* mutant was carried out in the same process as that described for the *iifR* mutant. Overlapping PCR was used to create an *iif* promoter region and *iifC* gene fusion. The *iifC* gene was amplified by performing PCR using the *A. baumannii* ATCC 19606 chromosome DNA as template and the cIifCF and cIifCR primers. The *iif* promoter region was amplified with cIifPF and cIifPR primers. These two PCR products were purified and ligated by overlapping PCR using cIifPF and cIifCR primers. The final PCR product was cloned into the pJET1.2 vector (Fermentas) to generate the pJCiifC plasmid. The *Bam*HI fragment containing *iif* promoter and *iifC* gene from pJCiifC was isolated and then ligated into *Bam*HI-digested pMMB67EH, generating the complementing plasmid pComIifC. pComIifC was electroporated into the *A. baumannii* *iifC* mutant and transformants were selected on LB agar containing ampicillin (500 µg/ml).

**References**

1. Alexeyev MF, Shokolenko IN (1995) Mini-Tn*10* transposon derivatives for insertion mutagenesis and gene delivery into the chromosome of gram-negative bacteria. Gene 160: 59-62.

2. Furste JP, Pansegrau W, Frank R, Blocker H, Scholz P, Bagdasarian M, Lanka E (1986) Molecular cloning of the plasmid RP4 primase region in a multi-host-range *tacP* expression vector. Gene 48:119-131.

3. Penwell WF, Arivett BA, Actis LA (2012) The *Acinetobacter baumannii* *entA* gene located outside the acinetobactin cluster is critical for siderophore production, iron acquisition and virulence. PLoS One 7: e36493.
