# Supplementary material for: Detoxification of Indole by an Indole-Induced Flavoprotein Oxygenase from Acinetobacter baumannii
Source: PLoS One. 2015 Sep 21;10(9):e0138798. doi: 10.1371/journal.pone.0138798 (PMC4577076; doi:10.1371/journal.pone.0138798)
Supplement: S1 Table — (DOCX) [file pone.0138798.s009.docx]

**S1 Table. The <KAN-2> transposon insertion sites within *iifC*.**

| Clone | Insertion site^a^ |
| --- | --- |
| A03 | 645 |
| H07 | 427 |
| B10 | 1153 |
| F01 | 400 |
| F02 | 45 |
| H08 | 139 |
| D08 | 653 |
| G04 | 335 |
| H04 | 675 |

^a^The insertion sites in relation to the translational start codon of *iifC* were determined by sequencing. The <KAN-2> insertion into target DNA creates 9-bp target-site duplication.
