# Supplementary material for: Detoxification of Indole by an Indole-Induced Flavoprotein Oxygenase from Acinetobacter baumannii
Source: PLoS One. 2015 Sep 21;10(9):e0138798. doi: 10.1371/journal.pone.0138798 (PMC4577076; doi:10.1371/journal.pone.0138798)
Supplement: S2 Table — (DOCX) [file pone.0138798.s010.docx]

**S2 Table. Purification of recombinant IifC.**

| Purification  step | Total protein | Total activity^*^ | Specific  activity | Purification |
| --- | --- | --- | --- | --- |
|  | *mg* | *units* | *units/mg* | *fold* |
| Crude extract | 1324 | 252 | 0.19 | 1 |
| Q-Sepharose HP | 602 | 2023 | 3.36 | 17.68 |

^*^ One unit was defined as the formation of one micromole of indoxyl per minute.
