# Supplementary material for: Detoxification of Indole by an Indole-Induced Flavoprotein Oxygenase from Acinetobacter baumannii
Source: PLoS One. 2015 Sep 21;10(9):e0138798. doi: 10.1371/journal.pone.0138798 (PMC4577076; doi:10.1371/journal.pone.0138798)
Supplement: Supplementary file 6 [file pone.0138798.s001.docx]

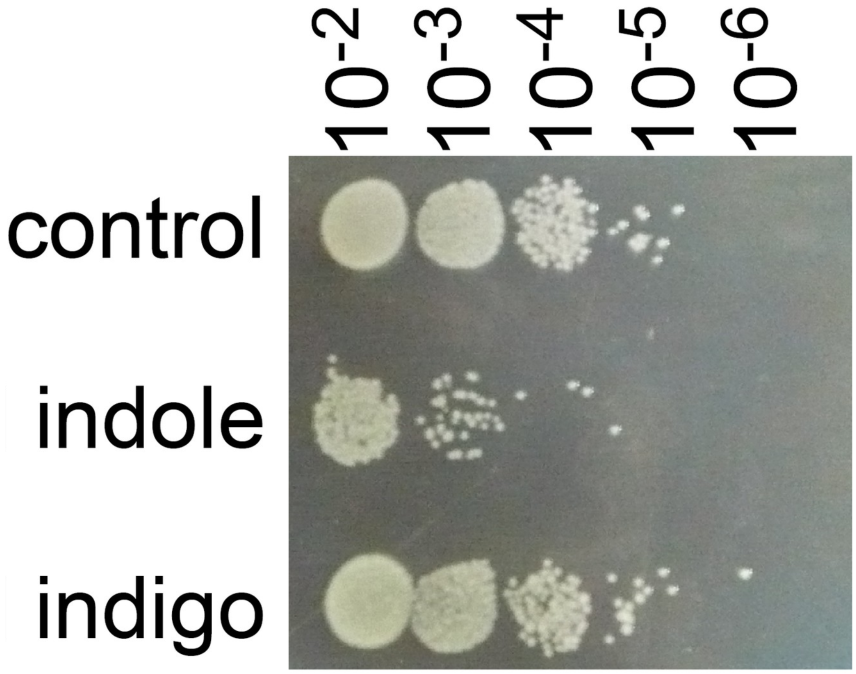
10-2
10-3
10-4
10-5
10-6
control
indole
indigo
